# ELK1 inhibition alleviates amyloid pathology and memory decline by promoting the SYVN1-mediated ubiquitination and degradation of PS1 in Alzheimer’s disease

**DOI:** 10.1038/s12276-025-01455-8

**Published:** 2025-05-01

**Authors:** Lilin Yi, Junjie Li, Yan He, Jiaojiao Wang, Maoju Wang, Song Guo, Man Luo, Bin Wu, Mingliang Xu, Qiuyun Tian, Yepeng Fan, Mulan Chen, Boqing Xu, Lei Xia, Weihong Song, Guiqiong He, Yehong Du, Zhifang Dong

**Affiliations:** 1 Growth, Development, and Mental Health of Children and Adolescence Center, Pediatric Research Institute, Ministry of Education Key Laboratory of Child Development and Disorders, National Clinical Research Center for Child Health and Disorders, Chongqing Key Laboratory of Child Neurodevelopment and Cognitive Disorders, Children’s Hospital of Chongqing Medical University, Chongqing, China; 2 Townsend Family Laboratories, Department of Psychiatry, University of British Columbia, Vancouver, British Columbia Canada; 3 Oujiang Laboratory (Zhejiang Lab for Regenerative Medicine, Vision and Brain Health), Institute of Aging, Key Laboratory of Alzheimer’s Disease of Zhejiang Province, Zhejiang Clinical Research Center for Mental Disorders, School of Mental Health and The Affiliated Kangning Hospital, Wenzhou Medical University, Wenzhou, China; 4 Department of Anatomy, Basic Medical College, Chongqing Medical University, Chongqing, China

**Keywords:** Hippocampus, Ubiquitylation

## Abstract

ELK1 is a member of the E-twenty-six transcription factor family and is usually activated by phosphorylation at Ser383 and Ser389 by extracellular signal-regulated kinase 1/2 (ERK1/2). Dysregulation of ERK1/2 is involved in Alzheimer’s disease (AD)-related neuropathogenesis and cognitive impairments. However, the role of ELK1 in AD pathogenesis remains unclear. Here we report that the expression of ELK1 was significantly increased in the brain tissues of patients with AD and AD model mice. The genetic knockdown of ELK1 or inhibition of its phosphorylation by an interfering peptide (TAT-DEF-ELK1 (TDE)) reduced amyloidogenic processing of APP by targeting PS1, consequently inhibiting Aβ generation and alleviating synaptic and memory impairments in APP23/PS45 double-transgenic AD model mice. In addition, we further found that ELK1 regulated the expression of PS1 by competitively inhibiting the interaction between PS1 and its E3 ubiquitin ligase synoviolin (SYVN1), thereby inhibiting the SYVN1-mediated ubiquitination and degradation of PS1. Our results demonstrate that ELK1 aberrantly increases in AD and genetic or pharmacological inhibition of ELK1 can alleviate AD-related pathology and memory impairments by enhancing the SYVN1-mediated PS1 ubiquitination and degradation, indicating that ELK1 may be a novel target for AD treatment.

## ELK1 elevation linked to Alzheimer’s disease athology

Alzheimer’s disease is a leading cause of memory impairment in older adults. Researchers found that ELK1 levels are higher in patients with Alzheimer’s disease and animal models. Through genetic and drug methods to suppress ELK1 activity, they observed reduced accumulation of pathogenic proteins and improved memory in mice. This study employs a comprehensive approach involving human and murine brain tissues analysis, as well as cell cultures. The investigation specifically examined the regulatory effects of ELK1 on PS1, a protein implicated in the production of neurotoxic amyloid plaques. They discovered that ELK1 inhibits PS1 degradation, leading to more plaque formation. By suppressing ELK1 activity, they observed a decrease in plaque formation and improvements in memory tests for mice. This suggests that targeting ELK1 could be a potential treatment strategy for Alzheimer’s disease.

This summary was initially drafted using artificial intelligence, then revised and fact-checked by the author.

## Introduction

Alzheimer’s disease (AD) is the foremost cause of dementia among the elderly^1,2^. It is marked by the presence of extracellular senile plaques formed through the accumulation of amyloid-β (Aβ) peptides, intracellular neurofibrillary tangles composed of abnormally hyperphosphorylated tau protein, alongside the loss of synapses and neurons^3^. Aβ peptides, the primary constituents of senile plaques, are generated through the sequential cleavage of amyloid precursor protein (APP) by β-secretase (BACE1) and γ-secretase^4–6^. Initially, BACE1 cleaves APP to produce 89- or 99-residue transmembrane C-terminal fragments (β-CTFs, C89 or C99), which are subsequently processed by γ-secretase to yield Aβ peptides along with the APP intracellular domain^7–10^. The γ-secretase complex comprises four subunits: presenilin 1 or 2 (PS1 or PS2), nicastrin (NCT), presenilin enhancer 2 (PEN2) and anterior pharynx-defective phenotype 1 (APH1), with presenilin playing a crucial role in catalyzing the cleavage of substrates to generate Aβ peptides^11–13^.

Extracellular signal-regulated kinase 1/2 (ERK1/2), a vital component of the mitogen-activated protein kinase (MAPK) signaling cascade, functions as a serine/threonine protein kinase with significant implications in both physiological and pathological processes. Under physiological conditions, ERK1/2 activation plays an essential role in the development of the central nervous system^14–16^, maintenance of synaptic plasticity^17–21^, as well as the processes underlying learning and memory^19,22–27^. However, ERK1/2 is aberrantly overactivated in the brains of patients with AD and mouse models, which contributes to tau hyperphosphorylation, Aβ accumulation and subsequent synaptic and cognitive dysfunctions^28–31^. Conversely, inhibiting this overactivation with U0126, a specific inhibitor of ERK1/2, has been shown to reduce Aβ generation and improve synaptic function in AD models^32,33^. Collectively, these research advances show that ERK1/2 hyperactivation occurs and aggravates the progress of AD. However, the downstream molecular mechanism of ERK1/2 in the process of AD remains largely elusive.

E-twenty-six (ETS)-like protein 1 (ELK1), a member of the ternary complex factor subfamily of ETS-domain transcription factors^34^, is one of the most thoroughly studied targets of ERK1/2 and is typically activated via phosphorylation at Ser383 and Ser389^35,36^. Numerous studies have established ELK1’s involvement in cancer progression by regulating cellular proliferation, differentiation and survival^34,37^. However, despite its widespread expression across various brain regions such as the hippocampus, cortex, striatum and cerebellum^38^, the specific neurological functions of ELK1 within the central nervous system have not been extensively understood. As a transcription factor, ELK1 is present not only in the nucleus but also in neuronal soma, dendrites and axon terminals^38,39^, which indicates that ELK1 may have broader biological roles beyond its nuclear activity. Indeed, overexpression of ELK1 has been shown to reduce cell viability and promote apoptosis via interaction with mitochondrial permeability transition pore complex, whereas its genetic knockdown enhances cell viability in primary rat hippocampal neurons^40^. Moreover, ELK1 expression markedly increases in primary cortical neurons exposed to oxygen–glucose deprivation, and its inhibition confers notable neuroprotective effects against oxygen–glucose deprivation-induced neuronal damage^41^. More importantly, both ELK1 and its phosphorylated form (p-ELK1) might be linked to several human neurodegenerative diseases, including Parkinson’s disease, Huntington’s disease and AD^42^. However, the exact roles and molecular mechanisms of ELK1 in neurodegenerative diseases, especially in AD, remain unclear.

In this study, we observe a notable elevation in ELK1 protein levels in AD patients, model mice and model cells. Both genetic knockdown and pharmacological inhibition of ELK1 were found to reduce APP amyloidogenic processing by enhancing the SYVN1-mediated ubiquitination and degradation of PS1, leading to a reduction in senile plaque formation and an improvement in synaptic and cognitive function in AD model mice.

## Materials and methods

### Ethics approval and consent to participate

The human study was evaluated and approved by the Ethics Committee of Zhejiang University (Research Project Ethics approval document no. 2018-009). All animal experiments were performed in accordance with the Chongqing Science and Technology Commission guidelines and approved by the Animal Ethics Committee of Children’s Hospital of Chongqing Medical University (approval no. CHCMU-IACUC20210114017).

### Human samples

Human brain tissues were sourced from the Chinese Human Brain Bank of Zhejiang University, with detailed information provided in Supplementary Table 1.

### Animals

APP23/PS45 double-transgenic mice and their wild-type (WT) littermates (C57BL/6J) were bred and reared in a temperature and humidity controlled specific pathogen-free room at the Children’s Hospital of Chongqing Medical University. The mice were kept under a 12-hour light–dark cycle (lights on from 7:00 to 19:00) with free access to food and water. The genotypes of the mice were identified via PCR using DNA extracted from clipped tails.

### Antibodies

Anti-C20 (1:1,000) antibody used to detect APP and its β-CTFs was kindly provided by the lab of Professor Weihong Song. Anti-PS1 (1:1,000, ab76083) antibody used to detect PS1 holoprotein (PS1-FL) and PS1 C-terminal fragments(PS1-CTF) was purchased from Abcam. Anti-PS1 (1:500, R382308) antibody used to detect PS1 N-terminal fragments (PS1-NTF) was obtained from ZEN BIO. Anti-ELK1 (1:500, ab32106), anti-NCT (1:1,000, ab3444) and anti-SYVN1 (1:500, ab170901) antibodies were purchased from Abcam. Anti-BACE1 (1:1000, #5606), anti-PEN2 (1:1000, #8598), anti-HA-Tag (1:1000, #3724) and anti-LC3 (1:1,000, #12741) antibodies were sourced from Cell Signaling Technology. Anti-APH1A (1:1,000, NB100-74360) antibody was purchased from Novus Biologicals. Anti-p-ELK1 Ser383 (1:500, AF3212) antibody was obtained from Affinity Biosciences. Anti-ubiquitin (1:1,000, #10201-2-AP) and anti-NEDD4L (1:1,000, #13690-1-AP) antibodies were purchased from Proteintech. Anti-p62 (1:1000, H00008878-M01) antibody was purchased from Abnova. Anti-Flag-Tag (1:1,000, #3900002) antibody was obtained from ZEN BIO. Anti-β-actin (1:3,000, A5411) antibody was purchased from Sigma. Anti-β-amyloid antibody (clone 4G8) (1:500, #800705) was obtained from BioLegend.

### Plasmids

The plasmids utilized for mouse ELK1 overexpression and interference with ELK1 expression via small hairpin RNA were synthesized by Youze Biotechnology, while those employed for human ELK1 overexpression and interference with ELK1 expression were constructed by Genechem. Youze Biotechnology also provided plasmids for mouse Flag-tagged PS1 (PS1-Flag), HA-tagged ELK1 (ELK1-HA), HA-tagged SYVN1 (SYVN1-HA), HA-tagged WT ubiquitin (Ub-HA) and HA-tagged mutant ubiquitin (UbR-HA), in which all lysine (K) residues were replaced by arginine (R) residues and Flag-tagged PS1 mutants K311, K314, K380, K395, K429 and K430, where only the numbered K residue was retained, while the other K residues were mutated into R residues. HA-tagged ubiquitin mutants K6R, K11R, K27R, K29R, K33R, K48R and K63R, in which only the numbered K residue was mutated into R residue, while the other six K residues in ubiquitin were retained, were constructed by homologous recombination using Ub-HA as the template. HA-tagged ubiquitin mutants K27, K48 and K63, where only the numbered K residue was retained, while the other six K residues in ubiquitin were mutated into R residues, were generated by homologous recombination using UbR-HA as the template. A phosphorylation mimic plasmid of ELK1 Ser383/389 (p-ELK1) was created by homologous recombination using ELK1-HA as the template, mutating Ser383 and Ser389 to aspartic acid. The sequences of the primers used to construct mutant plasmids are provided in Supplementary Table 2.

### Cell culture and transfection

Mouse neuroblastoma N2A cells were grown in 47% Dulbecco’s modified Eagle’s medium (DMEM) (Gibco) and 47% Opti-MEM (Gibco) supplemented with 5% fetal bovine serum (FBS) (Gibco) and 1% penicillin–streptomycin (Gibco). The N2A^APP^ cells were grown in DMEM supplemented with 10% FBS, 1% penicillin–streptomycin and 50 µg ml^−1^G418 (Gibco). The human embryonic kidney (HEK293) cells were grown in DMEM supplemented with 10% FBS and 1% penicillin–streptomycin. The 2EB2 cells were grown in DMEM containing 10% FBS, 1% penicillin–streptomycin, 25 µg ml^−1^G418 and 100 µg ml^−1^zeocin (Invitrogen). All cell lines were maintained at 37 °C in a humidified atmosphere containing 5% CO_2_. Transfection experiments were performed using Lipofectamine 3000 Transfection Reagent (Invitrogen) following the manufacturer’s protocol. Briefly, when the cell confluence reached approximately 70%, the complete medium was substituted with serum-free medium. The plasmids and P3000 reagent were diluted in Opti-MEM and Lipofectamine 3000 was also diluted in Opti-MEM. Then, the diluted plasmids and the diluted Lipofectamine 3000 were thoroughly mixed. Following 15-min incubation, the mixtures were added to wells, with cells subsequently cultured for another 24–48 h.

To block the proteasomal or lysosomal pathways, the N2A^APP^ cells were exposed to 10 μM MG132 (MCE) or 50 μM chloroquine (CQ) (Sigma) for 24 h^43^. To assess Aβ-induced ELK1 and p-ELK1 expression, the N2A and HEK293 cells were treated with varying concentrations of Aβ_1-42_ peptide (GL Biochem) for 24 h. To examine the effect of SYVN1 inhibition on PS1 expression, the N2A^APP^ cells were incubated with different concentrations of SYVN1-specific inhibitor LS-102 (MCE) for 48 h.

For experiments assessing PS1 degradation, the cells were transfected with plasmids overexpressing ELK1 or SYVN1 for 4–6 h. Following this, the cells were exposed to 100 μg ml^−1^cycloheximide (CHX) (CST) for different time points^32^.

### Adeno-associated virus and microinjection

To knock down ELK1 in vivo, adeno-associated virus carrying ELK1 small hairpin RNA (AAV_shELK1_) with a titer of 3 × 10^12^ TU ml^−1^was created by OBiO Technology. The mice were anesthetized by intraperitoneal (i.p.) injection of sodium pentobarbital (60 mg kg^−1^) and then fixed on a stereotaxic device. After the coordinates were determined, 1 μl AAV_shELK1_ was microinjected into the hippocampal CA1 region through a drilled opening (−2.5 mm posterior, ±2.0 mm lateral and −2.5 mm ventral relative to bregma). The mice received AAV microinjections at 2 months of age and were subjected to behavioral assessments at 5 months.

### Peptide synthesis and administration

The TAT-DEF-ELK1 (TDE) peptide (GRKKRRQRRRPPSPAKLSFQFPSSGSAQVHI) and its scramble (GRKKRRQRRRPPQSKPSGSQHPIFSLAFVAS)^44^ were synthesized by GL Biochem. For in vivo administration, the peptides were dissolved in sterile saline. The mice were daily injected (i.p., 8 mg kg^−1^) with either TDE or scramble peptide from the age of 2 months to the end of the behavioral tests. The behavioral tests were conducted at the age of 5 months in mice. For in vitro assays, the cells were exposed to 20 μM TDE or scramble peptide to inhibit ELK1 phosphorylation.

### Barnes maze test

The Barnes maze test was conducted on an elevated (120 cm in height) white circular platform (75 cm in diameter). There were 18 evenly spaced holes (5 cm in diameter) along its edge, with one leading to an escape box. The platform was surrounded by light blue curtains decorated with three spatial cues of distinct geometrical configurations. A CCD camera positioned above the platform captured the tracking data, which was analyzed using the ANY-maze video tracking system (Stoelting). To familiarize the mice with the maze, 1 day before spatial training, we placed the mice at the platform center and enabled them to explore freely for 3 min. Over the following 5 days, each mouse underwent spatial learning task twice a day. During each trial, the mice started from the platform center and had up to 3 min to locate the escape box. If they were unable to locate the box, they would be gently guided to it and remain there for 1 min. 1 day following the last learning task, a probe test was conducted with the escape box removed. In this test, mice were again placed at the platform center and permitted a 3-min free exploration.

### Morris water maze test

The Morris water maze test was performed in a circular pool (150 cm in diameter, 50 cm in height). The pool was filled with water clouded by nontoxic white paint. The water was kept at 23–25 °C to make it suitable for the mice to swim and explore. Surrounding the pool were light blue curtains featuring three uniquely shaped spatial cues. A CCD camera positioned above the pool captured the tracking data, which was analyzed using the ANY-maze video tracking system. To familiarize the mice with the maze, 1 day before spatial training, the mice were permitted to swim freely in the pool for 2 min. During the acquisition training, a hidden platform (also called island, 7.5 cm in diameter) was positioned 1 cm below the water surface. Over the 5 days of acquisition training, each mouse underwent spatial learning task four trials a day. Each trial began with themice being put into the water from one of four starting points (northeast [NE], northwest [NW], southwest [SW], andsoutheast [SE]) while they faced the pool wall. Each mouse was given up to 2 min to locate the platform. If they were unable to locate the platform, they would be guided to it and remain there for 20 s. 1 day following the last learning task, a probe test was performed with the hidden platform removed. In this test, the mice were placed in the pool at the SW starting point and permitted a 2-min free exploration.

### Immunohistochemistry and immunofluorescence

After behavioral tests, the mice were deeply anesthetized with urethane (1.5 g kg^−1^, i.p.) and subsequently transcardially perfused with phosphate-buffered saline until the liver turned white. The mouse brains were dissected and postfixed in freshly prepared 4% paraformaldehyde for 48 h followed by dehydration in 30% sucrose until the tissue sank. Subsequently, the coronal brain sections (30 μm thick) were prepared by embedding the tissue in optimal cutting temperature compound and sectioning with a cryostat.

For immunohistochemical assay, the brain coronal frozen sections were first treated with 88% formic acid for 15–30 min. Subsequently, the sections underwent a 30-min incubation in 30% hydrogen peroxide (H_2_O_2_). After blocking with 10% bovine serum albumin for 1 h at room temperature, the sections were incubated overnight at 4 °C with 4G8 antibody (1:500, BioLegend). The plaques were detected using the ABC-DAB method, and all the stained slides were scanned with a whole-slide scanner (Olympus). The number of plaques was quantified as previously described^45^.

For immunofluorescent assay, the brain sections were mounted on slides and dried at 60 °C for 45 min. Subsequently, the brain sections were blocked with 10% bovine serum albumin for 1 h, followed by overnight incubation with primary antibody at 4 °C. On the following day, the sections were incubated for 2 h with DAPI (1:1,000) and the appropriate fluorescent secondary antibody (1:1,000). After mounting with antifade medium, the sections were examined and imaged using a confocal microscope (Nikon).

### Western blot

The cells or brain tissues were lysed in lysis buffer supplemented with protease inhibitors and phosphatase inhibitors for 15–30 min. After thorough lysis, the lysates were centrifuged at 12,000 rpm for 15 min at 4 °C to collect the supernatants. The protein concentrations were measured using a BCA Protein Assay Kit (Thermo Fisher Scientific). Equal amounts of protein were denatured by boiling with 5× sample buffer at 95 °C for 5 min. Subsequently, the samples were separated by SDS–sulfate polyacrylamide gel electrophoresis and transferred onto an immobilon-PTM polyvinylidene difluoride membrane (Millipore). Following a 1.5-h blocking with 5% nonfat milk, the membranes were incubated overnight at 4 °C with primary antibodies. On the next day, the membranes underwent a 1.5-h incubation with the corresponding HRP-labeled secondary antibody (1:3,000, Perkin-Elmer). The protein bands were visualized using ECL substrate (Bio-Rad) and imaged with a Bio-Rad Imager (Bio-Rad). The relative expression levels of target proteins were calculated using Quantity One software (Bio-Rad) and normalized to β-actin.

### Aβ ELISA

The mouse hippocampal tissues were homogenized in lysis buffer supplemented with protease inhibitors for 15–30 min. After thorough lysis, the homogenates were centrifuged at 12,000 rpm for 15 min at 4 °C to collect the supernatants. The resulting samples were standardized to equal protein concentrations and subsequently diluted 40-fold for Aβ detection. Aβ_40_ and Aβ_42_ levels were quantitatively determined using the corresponding commercially available enzyme-linked immunosorbent assay (ELISA) kits (R&D) following the manufacturer’s guidelines. Briefly, samples or standards were added to a 96-well polystyrene microplate precoated with a monoclonal antibody specific for either human Aβ_40_ or Aβ_42_ and incubated for 2 h at 4 °C. The plate was then washed three to four times, followed by the addition of human Aβ_40_ or Aβ_42_ conjugate and a second 2-h incubation at 4 °C. After a subsequent wash cycle, substrate solution was added, and the plate was incubated for 30 min at room temperature away from light. The reaction was terminated with stop solution, and the absorbance at 450 nm was measured within 30 min. To ensure accuracy and precision, each sample was tested in duplicate wells.

### Coimmunoprecipitation

The cells were lysed in the lysis buffer designed for IP (Beyotime Biotechnology), which included protease inhibitors and phosphatase inhibitors. Following extensive lysis, the lysates were centrifuged at 12,000 rpm for 15 min at 4 °C to obtain the supernatants. After determination of protein concentrations using the BCA assay, 500–1,000 µg of protein was incubated overnight at 4 °C with primary antibodies or nonspecific IgG on a rotator. Following this, protein A/G magnetic beads for IP (Bimake) were added and incubated for another 2–3 h. The precipitates were washed three to five times with phosphate-buffered saline to remove nonspecific binding. The bound proteins were eluted from the precipitates by boiling in 1× SDS–sulfate polyacrylamide gel electrophoresis loading buffer at 95 °C for 5 min and subsequently prepared for western blot analysis.

### Electrophysiological recording

The mice were deeply anesthetized with urethane (1.5 g kg^−1^, i.p.) and then underwent transcardial perfusion with artificial cerebral spinal fluid (ACSF). The mouse brains were isolated and soaked in ice-cold ACSF oxygenated with 95% O_2_ and 5% CO_2_. Coronal hippocampal sections, each 400 μm thick, were prepared using a vibratome (VT1200S, Leica). These sections were immersed in ACSF with 95% O_2_ and 5% CO_2_ for 2 h at 35 °C. Field excitatory postsynaptic potential (fEPSP) in the hippocampal CA1 stratum radiatum was recorded by stimulating the Schaffer collateral–commissural pathway. After establishing a stable baseline, a theta burst stimulation was applied to induce long-term potentiation (LTP). The data acquisition was performed with the PatchMaster v2.73 software (HEKA Electronic, Lambrecht/Pfalz).

### Statistics

Statistical analysis is conducted with SPSS 22.0 software. The results are presented as mean ± standard error of the mean, with appropriate analyses conducted via one-way analysis of variance (ANOVA), repeated measures two-way ANOVA or two-tailed Student’s *t*-tests. *P* < 0.05 is considered statistically significant.

## Results

### The expression of ELK1 is increased in AD

**Fig. 1:**
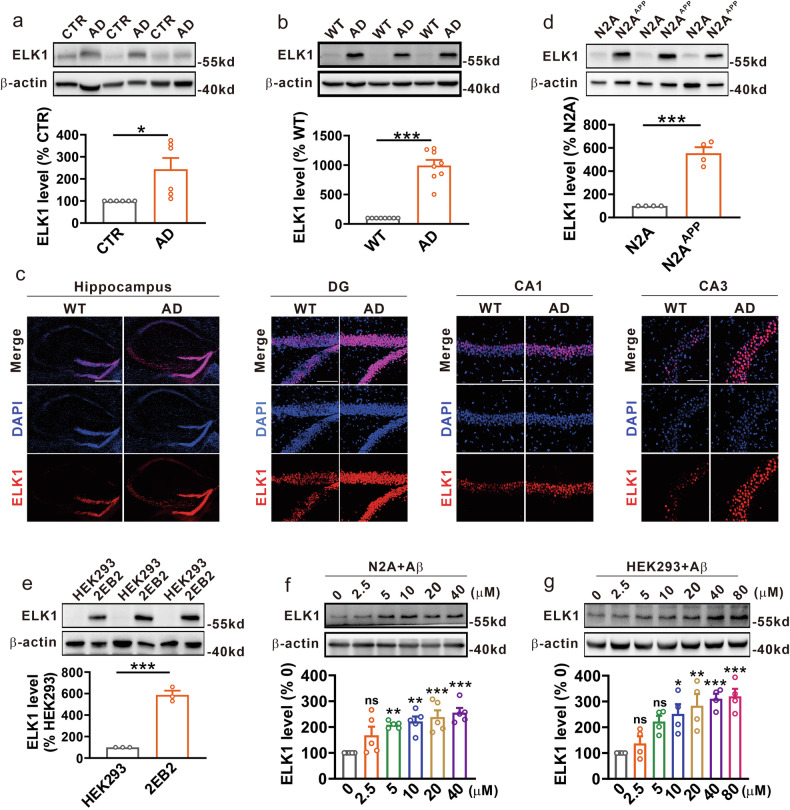
ELK1 was increased in AD. **a** Western blot (WB) of ELK1 in hippocampus of patients with AD and controls (CTR) (*n* = 6 in each group). *P < 0.05, determined by an unpaired Student’s *t*-test. **b** WB of ELK1 in hippocampus of WT and AD mice at 6 months (*n* = 8 in each group). ***P < 0.001, determined by an unpaired Student’s *t*-test. **c** Immunofluorescence of ELK1 in different hippocampal regions of WT and AD mice at 6 months. Scale bars, 500 μm for hippocampus and 100 μm for DG, CA1 and CA3 (*n* = 4 in each group). **d** WB of ELK1 in N2A and N2A^APP^ cells (n = 4 in each group). ***P < 0.001, determined by an unpaired Student’s *t*-test. **e** WB of ELK1 in HEK293 and 2EB2 cells (*n* = 3 in each group). ***P < 0.001, determined by an unpaired Student’s *t*-test. **f** WB of ELK1 in N2A cells treated with different concentrations of Aβ_1-42_ peptide (*n* = 5 in each group). **P < 0.01 and ***P < 0.001, determined by one-way ANOVA. **g** WB of ELK1 in HEK293 cells treated with different concentrations of Aβ_1-42_ peptide (*n* = 4 in each group). *P < 0.05, **P < 0.01 and ***P < 0.001, determined by one-way ANOVA. ns, not significant.

To assess changes in ELK1 expression associated with AD, brain tissue from patients with AD and control individuals was examined. Our findings revealed a significant elevation in ELK1 protein levels within the hippocampus of patients with AD when compared with age-matched controls (Fig. 1a). Subsequently, we detected ELK1 expression in the APP23/PS45 double-transgenic mouse model of AD. Similar to the findings in human patients, ELK1 protein levels were notably elevated in the hippocampus of 6-month-old AD model mice relative to WT littermates (Fig. 1b). Immunofluorescence staining further demonstrated a significant rise in ELK1 expression within the hippocampal regions (DG, CA1 and CA3) of AD model mice relative to WT mice (Fig. 1c). In addition, we evaluated ELK1 expression in AD model cells, specifically N2A cells stably expressing human Swedish mutant APP695 (N2A^APP^) and HEK293 cells stably transfected with the same mutant APP695 along with BACE1 (2EB2). The results indicated a marked increase in ELK1 protein levels in both N2A^APP^ and 2EB2 cells relative to their respective controls, N2A and HEK293 cells (Fig. 1d, e). Meanwhile, we analyzed ELK1 expression in acute cellular models of AD induced by the Aβ_1-42_ peptide treatment. The results showed a concentration-dependent increase in ELK1 expression with Aβ exposure in both N2A and HEK293 cells (Fig. 1f, g). Taken together, these results indicate a clear upregulation of ELK1 protein in various contexts of AD, encompassing patients, model mice and model cells.

### ELK1 promotes APP amyloidogenic processing by inhibiting the ubiquitination and degradation of PS1

**Fig. 2:**
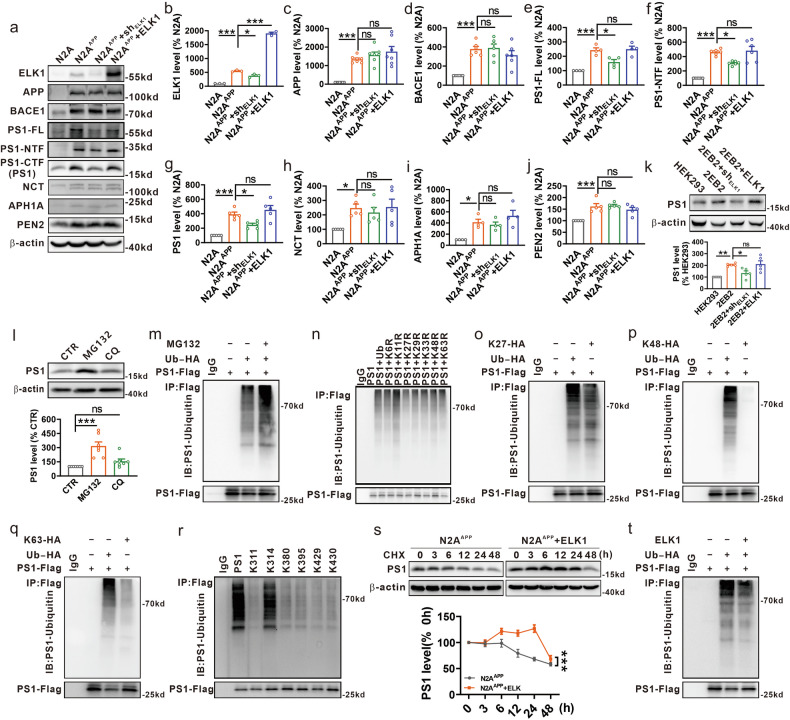
ELK1 promoted APP amyloidogenic processing by inhibiting the ubiquitination and degradation of PS1. **a** ELK1 or ELK1 small hairpin RNA (sh_ELK1_) plasmid was transfected to N2A^APP^ cells. **a**–**j** ELK1 (**a**, **b**), APP (**a**, **c**), BACE1 (**a**, **d**), PS1 (**a**, **e**–**g**), NCT (**a**, **h**), APH1A (**a**, **i**) and PEN2 (**a**, **j**) were determined by western blot (WB) 48 h later (*n* = 3–7 in each group). *P < 0.05 and ***P < 0.001, determined by one-way ANOVA. **k** ELK1 or sh_ELK1_ plasmid was transfected to 2EB2 cells. PS1 was determined by WB 48 h later (*n* = 5 in each group). *P < 0.05 and **P < 0.01, determined by one-way ANOVA. **l** The N2A^APP^ cells were exposed to MG132 (10 μM) or CQ (50 μM). PS1 was determined by WB 24 h later (*n* = 7 in each group). ***P < 0.001, determined by one-way ANOVA. **m** The HEK293 cells were treated with or without MG132. PS1**-**Flag together with or without Ub-HA plasmid was transfected to these cells 6 h later. PS1 ubiquitination was evaluated 24 h later by immunoprecipitation (IP) with antibodies to Flag or negative control antibodies (IgG) and immunoblotting (IB) with antibodies to HA (*n* = 3 in each group). **n** PS1-Flag together with Ub-HA or HA-tagged ubiquitin mutants (K6R, K11R, K27R, K29R, K33R, K48R and K63R) was transfected to HEK293 cells. PS1 ubiquitination was evaluated by coimmunoprecipitation (Co-IP) as described above (*n* = 2 in each group). **o**–**q** PS1-Flag together with Ub-HA or HA-tagged ubiquitin mutants K27 (**o**), K48 (**p**) and K63 (**q**) was transfected to HEK293 cells. PS1 ubiquitination was evaluated by Co-IP as described above (*n* = 2–3 in each group). **r** PS1-Flag or Flag-tagged PS1 mutants (K311, K314, K380, K395, K429 and K430) was transfected to HEK293 cells. The ubiquitination of PS1 and PS1 mutants were evaluated 24 h later by IP with antibodies to Flag or IgG and IB with antibodies to ubiquitin (*n* = 4 in each group). **s** The N2A^APP^ cells were transfected with or without ELK1, followed by exposure to CHX (100 μg ml^−1^) for the indicated time. PS1 was determined by WB to evaluate its degradation (*n* = 8 in each group). ***P < 0.001, determined by two-way ANOVA. **t** PS1-Flag and Ub-HA, along with or without ELK1, were transfected to HEK293 cells. PS1 ubiquitination was evaluated by Co-IP as described above (*n* = 3 in each group). ns, not significant.

To investigate the role of ELK1 in APP processing, N2A^APP^ cells were transfected with ELK1 or ELK1 small hairpin RNA (sh_ELK1_) plasmid to induce overexpression or knockdown of ELK1, respectively (Fig. 2a, b). Neither knockdown nor overexpression of ELK1 affected the expression of APP (Fig. 2a, c) and BACE1 (Fig. 2a, d). Notably, knockdown of ELK1 led to a significant reduction in the γ-secretase component PS1, including PS1 holoprotein (PS1-FL), PS1 N-terminal fragments (PS1-NTF) and PS1 C-terminal fragments (PS1-CTF) (Fig. 2a, e–g), while leaving other components such as NCT, APH1A and PEN2 unaffected (Fig. 2a, h–j). Conversely, overexpression of ELK1 had no effect on the expression of all γ-secretase complex proteins (Fig. 2a, e–j). It has been well documented that PS1 undergoes endoproteolytic cleavage, generating N-terminal and C-terminal fragments (PS1-NTF and PS1-CTF, respectively) that assemble into functional PS1 heterodimers, which are essential for γ-secretase activation^46,47^. Our study revealed that ELK1 interacted specifically with PS1-CTF but not with PS1-NTF (Supplementary Fig. 1). Therefore, we next focused on the relationship between ELK1 and PS1-CTF. Unless otherwise specified, PS1 detected in the present study refers to PS1-CTF. In agreement with the results in N2A^APP^ cells, PS1 levels were notably elevated in 2EB2 cells compared with HEK293 cells (Fig. 2k). ELK1 knockdown significantly lowered PS1 levels, while ELK1 overexpression did not change PS1 levels in 2EB2 cells (Fig. 2k and Supplementary Fig. 2). Collectively, these data suggest that ELK1 may affect APP processing by regulating PS1.

ELK1 usually functions as a transcription factor, influencing the expression of specific target genes^34^. Therefore, we sought to investigate whether ELK1 modulates PS1 gene transcription in AD. Our analysis indicated that neither knockdown nor overexpression of ELK1 affected PS1 messenger RNA (mRNA) levels in 2EB2 cells (Supplementary Fig. 3c), indicating that ELK1 does not regulate PS1 gene transcription. Since ELK1 did not affect the synthesis of PS1, we then aimed to examine whether ELK1 affects the degradation of PS1 protein. It is well established that the ubiquitin–proteasome system and the autophagy–lysosome system are the primary mechanisms for protein degradation in eukaryotic cells^48^. To clarify the degradation route of PS1, we examined PS1 expression in N2A^APP^ cells subjected to treatment with proteasome inhibitor MG132 (10 μM for 24 h) or autophagy inhibitor CQ (50 μM for 24 h) (Supplementary Fig. 4). The data revealed a notable increase in PS1 levels in N2A^APP^ cells treated with MG132 rather than CQ, compared with untreated controls (Fig. 2l), indicating that PS1 was predominantly degraded by the ubiquitin–proteasome pathway. To further validate this, the HEK293 cells were cotransfected with flag-tagged PS1 (PS1-Flag) and HA-tagged ubiquitin (Ub-HA) plasmids followed by treatment with or without MG132. Immunoprecipitation and subsequent western blot analysis confirmed that PS1 was indeed poly-ubiquitinated, with both total and ubiquitinated PS1 levels markedly elevated following MG132 treatment (Fig. 2m).

Ubiquitin contains seven lysine (K) residues, namely K6, K11, K27, K29, K33, K48 and K63, each capable of forming internal conjugations to generate polyubiquitination chains on substrates. To discern which polyubiquitination chain is associated with PS1, we cotransfected HEK293 cells with PS1-Flag and either Ub-HA or HA-tagged ubiquitin mutants (K6R, K11R, K27R, K29R, K33R, K48R and K63R), where only the specified K residue was substituted with an arginine (R), rendering it functionally inactive, while the remaining six K residues were preserved. Our results indicated that, in comparison to Ub, only the expression of K27R diminished the polyubiquitination of PS1. Conversely, other ubiquitin mutants did not exhibit a similar effect, suggesting that PS1 primarily undergoes ubiquitination through K27-linked polyubiquitination chains (Fig. 2n). Given that K48 is the most common ubiquitination chain, and K63 has previously been identified to be associated with PS1^49^, we further evaluated the effects of K27, K48 and K63 chains on PS1 ubiquitination. We cotransfected the HEK293 cells with PS1-Flag and Ub-HA or HA-tagged ubiquitin mutants (K27, K48 and K63), where only the specified K residue was retained, while the other six K residues were replaced with R residues. The results further confirmed that PS1 is predominantly ubiquitinated by K27-linked polyubiquitination chains (Fig. 2o–q). To pinpoint the ubiquitination sites on PS1, we generated a series of Flag-tagged PS1 lysine mutants, including K311, K314, K380, K395, K429 and K430. In these mutants, only the designated K residue was preserved, while the remaining K residues were mutated to R residues. Upon transfecting PS1-Flag or these mutants into HEK293 cells, we identified K314 as the primary ubiquitination site for PS1 (Fig. 2r).

Furthermore, we observed obviously delayed degradation of PS1 in N2A^APP^ cells transfected with ELK1 plasmid compared with its control, when CHX was administered to inhibit protein synthesis (Fig. 2s and Supplementary Fig. 5a). Moreover, ELK1 overexpression led to decreased levels of ubiquitinated PS1 alongside an accumulation of total PS1 in HEK293 cells cotransfected with PS1-Flag, Ub-HA and ELK1 plasmids (Fig. 2t and Supplementary Fig. 5b), indicating that ELK1 inhibited the ubiquitination and subsequent degradation of PS1.

### ELK1 inhibits the SYVN1-mediated ubiquitination and degradation of PS1

**Fig. 3:**
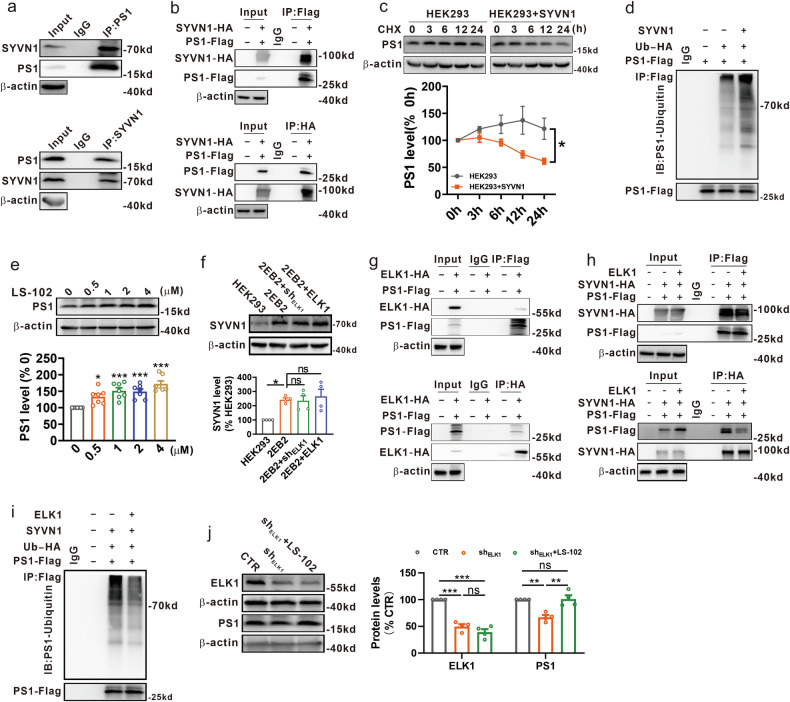
ELK1 inhibited the SYVN1-mediated ubiquitination and degradation of PS1. **a** Coimmunoprecipitation (Co-IP) of endogenous PS1 and SYVN1 in N2A^APP^ cells was performed using antibodies to PS1 and SYVN1 (*n* = 3 in each group). **b** Co-IP of exogenous PS1 and SYVN1 in HEK293 cells cotransfected with PS1-Flag and SYVN1-HA was performed using antibodies to Flag and HA (*n* = 3–4 in each group). **c** The HEK293 cells were transfected with or without SYVN1, followed by exposure to CHX (100 μg ml^−1^) for the indicated time. PS1 was determined by western blot (WB) to evaluate its degradation (*n* = 4 in each group). *P < 0.05, determined by two-way ANOVA. **d** PS1-Flag and Ub-HA, along with or without SYVN1, were transfected to HEK293 cells. PS1 ubiquitination was evaluated by Co-IP as described above (*n* = 3 in each group). **e** WB of PS1 in N2A^APP^ cells treated with different concentrations of LS-102 (*n* = 7 in each group). *P < 0.05 and ***P < 0.001, determined by one-way ANOVA. **f** ELK1 or sh_ELK1_ plasmid was transfected to 2EB2 cells. SYVN1 was determined by WB 48 h later (*n* = 4 in each group). *P < 0.05, determined by one-way ANOVA. **g** Co-IP of exogenous PS1 and ELK1 in HEK293 cells cotransfected with PS1-Flag and ELK1-HA was performed using antibodies to Flag and HA (*n* = 3 in each group). **h** PS1-Flag and SYVN1-HA, along with or without ELK1, were transfected to the HEK293 cells. Co-IP of exogenous PS1 and SYVN1 was performed as described above (*n* = 3–4 in each group). **i** PS1-Flag, Ub-HA and SYVN1, along with or without ELK1, were transfected to HEK293 cells. PS1 ubiquitination was evaluated by Co-IP as described above (*n* = 3 in each group). **j** WB of ELK1 and PS1 in N2A^APP^ cells transfected with sh_ELK1_, along with or without LS-102 treatment (1 μM for 48 h) (*n* = 4 in each group). **P < 0.01 and ***P < 0.001, determined by one-way ANOVA. ns, not significant.

Next, we wanted to determine the potential E3 ubiquitin ligase responsible for the ubiquitination and degradation of PS1. According to UbiBrowser predictions, SYVN1 is the most likely E3 ubiquitin ligase of PS1, followed by NEDD4L. To confirm the prediction, we detected the interactions between PS1 and either SYVN1 or NEDD4L. The results showed that only SYVN1 (Fig. 3a) but not NEDD4L (Supplementary Fig. 6) could interact with PS1 in N2A^APP^ cells. To further substantiate the interaction between PS1 and SYVN1, we transfected HEK293 cells with PS1-Flag and HA-tagged SYVN1 (SYVN1-HA) plasmids, which also demonstrated a clear interaction (Fig. 3b). More importantly, we found that SYVN1 overexpression significantly accelerated the degradation of PS1 (Fig. 3c and Supplementary Fig. 7a) through increasing the level of ubiquitinated PS1 (Fig. 3d and Supplementary Fig. 7b). In addition, LS-102, a selective SYVN1 inhibitor^50,51^, which was reported to inhibit polyubiquitination and degradation of SYVN1 target proteins^52,53^, led to a significant increase in the protein levels of PS1 (Fig. 3e). These results suggest that the SYVN1-mediated PS1 ubiquitination is critical to PS1 degradation.

The above results showed that SYVN1 is the E3 ubiquitin ligase of PS1, and ELK1 could inhibit PS1 ubiquitination. Subsequently, we sought to explore the involvement of SYVN1 in ELK1-mediated inhibition of PS1 ubiquitination. Our analysis revealed that neither knockdown nor overexpression of ELK1 influenced the levels of SYVN1, though SYVN1 was significantly increased in 2EB2 cells compared with HEK293 cells (Fig. 3f and Supplementary Fig. 2). However, it is unclear whether ELK1 regulates the interaction between PS1 and SYVN1 and thus affecting the SYVN1-mediated PS1 degradation. We observed the interaction between PS1 and ELK1 in HEK293 cells transfected with PS1-Flag and HA-tagged ELK1 (ELK1-HA) plasmids by coimmunoprecipitation (Fig. 3g). Notably, ELK1 overexpression significantly weakened the interaction between PS1 and SYVN1 in HEK293 cells (Fig. 3h). Moreover, overexpression of ELK1 markedly attenuated the SYVN1-mediated PS1 ubiquitination (Fig. 3i and Supplementary Fig. 7c). To further confirm the role of SYVN1 in sh_ELK1_-mediated PS1 ubiquitination and degradation, LS-102 was added to N2A^APP^ cells transfected with sh_ELK1_. As expected, LS-102 reversed the sh_ELK1_-induced reduction in PS1 expression (Fig. 3j). Collectively, these results suggest that ELK1 inhibits the interaction between PS1 and SYVN1, consequently reducing the SYVN1-mediated ubiquitination and degradation of PS1 in AD.

### Knockdown of ELK1 ameliorates synaptic and cognitive impairments in APP23/PS45 mice

**Fig. 4:**
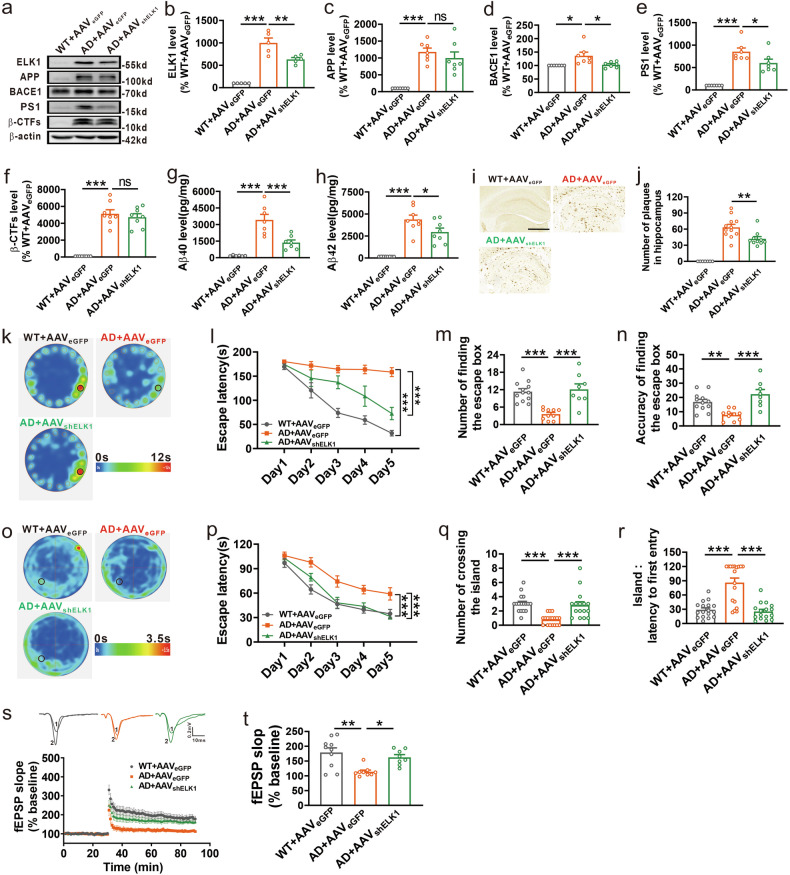
Knockdown of ELK1 alleviated synaptic and cognitive deficits in APP23/PS45 mice. **a** The mice were treated with different AAV microinjections at 2 months: WT mice administrated with AAV_eGFP_ (WT + AAV_eGFP_), APP23/PS45 mice administrated with AAV_eGFP_ (AD + AAV_eGFP_) and APP23/PS45 mice administrated with AAV_shELK1_ (AD + AAV_shELK1_). After a month of behavioral experiments which started at the age of 5 months, the mice were subjected to electrophysiological recordings or killed for molecular biological testing. **a**–**f** Western blot of ELK1 (**a**, **b**), APP (**a**, **c**), BACE1 (**a**, **d**), PS1 (**a**, **e**) and β-CTFs (**a**, **f**) in hippocampus of mice (*n* = 5–8 in each group). *P < 0.05, **P < 0.01 and ***P < 0.001, determined by one-way ANOVA. **g**, **h** Amounts of Aβ_40_ (**g**) and Aβ_42_ (**h**) measured by ELISA in hippocampus of mice (*n* = 6–8 in each group). *P < 0.05 and ***P < 0.001, determined by one-way ANOVA. **i**, **j** Senile plaques detected by immunohistochemistry in hippocampus of mice. Scale bars, 1 mm (*n* = 68–149 slices from 7–12 mice in each group). **P < 0.01, determined by one-way ANOVA. **k** The average heat map during memory retrieval in the Barnes maze test. **l** The latency to locate the escape box during spatial learning in the Barnes maze test (*n* = 8–12 in each group). ***P < 0.001, determined by a repeated measures ANOVA. **m** The number of finding the escape box during memory retrieval (*n* = 8–12 in each group). ***P < 0.001, determined by one-way ANOVA. **n** The accuracy of finding the escape box during memory retrieval (*n* = 8–12 in each group). **P < 0.01 and ***P < 0.001, determined by one-way ANOVA. **o** The average heat map during memory retrieval in the Morris maze test. **p** The latency for finding the island during spatial learning in the Morris maze test (*n* = 16–18 in each group). ***P < 0.001, determined by a repeated measures ANOVA. **q** The number of entries into the platform zone during memory retrieval (*n* = 16–18 in each group). ***P < 0.001, determined by one-way ANOVA. **r** The latency to first entry to platform area during memory retrieval (*n* = 16–18 in each group). ***P < 0.001, determined by one-way ANOVA. **s** The representative fEPSP traces and normalized slope plots (*n* = 8–10 slices from three to four mice in each group). **t** The bar graphs display the average percentage changes in fEPSP slope (*n* = 8–10 slices from three to four mice in each group). *P < 0.05 and **P < 0.01, determined by one-way ANOVA. ns, not significant.

Since knockdown of ELK1 reduced APP amyloidogenic processing in vitro (Fig. 2a–k), we aimed to investigate whether knockdown of ELK1 by adeno-associated virus carrying ELK1 shRNA (AAV_shELK1_) could alleviate amyloid pathology and cognitive deficits in APP23/PS45 double-transgenic model mice of AD. The mice received AAV_shELK1_ microinjection at 2 months of age, and spatial memory and amyloid pathology were measured at 5 months of age. Consistent with in vitro findings, levels of ELK1 and AD-associated proteins including APP, BACE1, PS1 and β-CTFs, were markedly increased in the hippocampus of AD model mice relative to WT controls (Fig. 4a–f). Importantly, AAV_shELK1_-mediated knockdown of ELK1 reversed the increases in BACE1 and PS1 but did not impact APP or β-CTFs levels in AD model mice (Fig. 4a–f). Since senile plaques resulting from Aβ peptide aggregation represent a major pathological hallmark of AD, we further evaluated the impact of ELK1 knockdown on Aβ generation and plaque formation. Consistent with expectations, both Aβ40 and Aβ42 levels, along with senile plaque counts, were markedly increased in the hippocampus of AD model mice relative to WT littermates (Fig. 4g–j). However, ELK1 knockdown led to a substantial decrease in Aβ40 and Aβ42 levels and the number of senile plaques (Fig. 4g–j).

The aforementioned results have revealed that knockdown of ELK1 could alleviate neuropathology in AD model mice. We then evaluated the influence of ELK1 knockdown on cognitive function through Barnes maze and Morris water maze tests. In the Barnes maze test, the AD model mice showed obvious spatial learning deficits compared with WT controls, characterized by prolonged escape latency to locate the escape box (Fig. 4l). The knockdown of ELK1 by AAV_shELK1_ significantly shortened the escape latency in AD model mice (Fig. 4l). In the subsequent probe test, the AD mice displayed impaired spatial memory retrieval, evidenced by a reduction in both the number (Fig. 4m) and accuracy (Fig. 4n) in exploring escape box. However, knockdown of ELK1 restored memory retrieval performance to control levels (Fig. 4m, n). Similarly, in the Morris water maze, the AD mice exhibited notable impairments in spatial learning, as AD mice took much longer to reach the hidden platform relative to WT controls (Fig. 4p). AAV_shELK1_-mediated ELK1 knockdown dramatically shortened the time required for AD mice to locate the hidden platform (Fig. 4p). In the probe test with the platform removed, the AD mice displayed impaired spatial memory retrieval, reflected by fewer platform crossings (Fig. 4q) and increased latency for initial entry into platform area (Fig. 4r) compared with WT mice. Remarkably, ELK1 knockdown restored both the number of platform crossings (Fig. 4q) and the latency for initial entry (Fig. 4r) to control levels in AD mice.

Given that LTP in the hippocampus is recognized as a fundamental mechanism for learning and memory, we detected the effect of ELK1 knockdown on LTP in the CA1 region of the hippocampus in AD model mice. We found that hippocampal LTP was markedly decreased in AD model mice relative to WT mice (Fig. 4s, t), whereas knockdown of ELK1 by AAV_shELK1_ reversed the impairment of LTP (Fig. 4s, t). Collectively, these data demonstrate that ELK1 knockdown mitigates neuropathology and alleviates synaptic and memory impairments in AD mice.

### ELK1 phosphorylation is involved in SYVN1-mediated ubiquitination and degradation of PS1

**Fig. 5:**
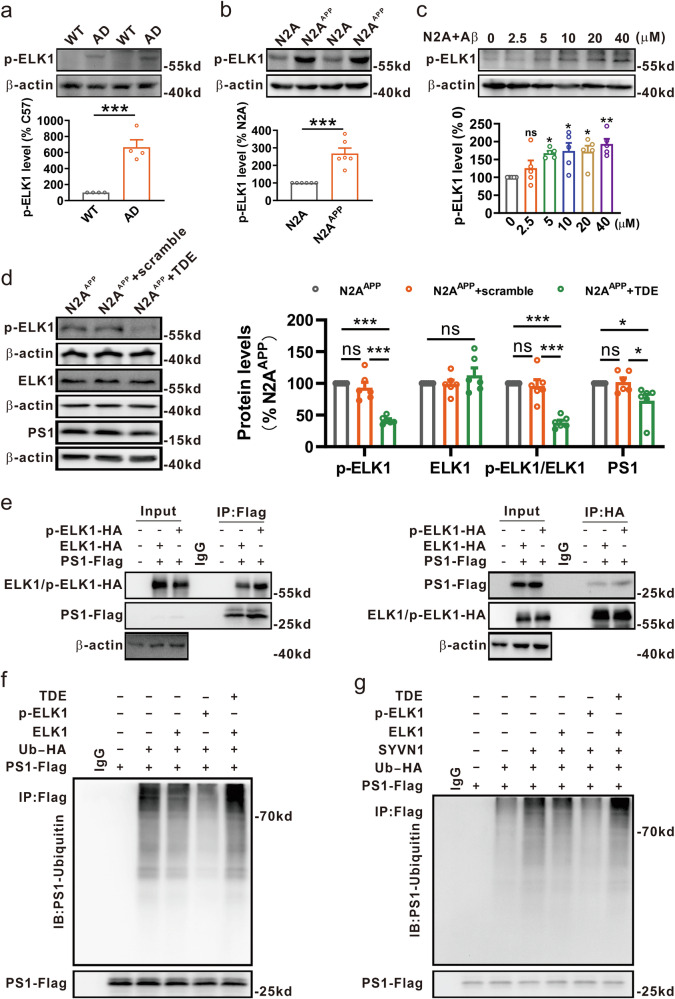
ELK1 phosphorylation inhibited the SYVN1-mediated ubiquitination and degradation of PS1. **a** Western blot (WB) of p-ELK1 in hippocampus of WT and AD mice at 6 months (*n* = 4 in each group). ***P < 0.001, determined by an unpaired Student’s *t*-test. **b** WB of p-ELK1 in N2A and N2A^APP^ cells (*n* = 6 in each group). ***P < 0.001, determined by an unpaired Student’s *t*-test. **c** WB of p-ELK1 in N2A cells treated with different concentrations of Aβ_1-42_ peptide (*n* = 5 in each group). *P < 0.05 and **P < 0.01, determined by one-way ANOVA. **d** WB of p-ELK1, ELK1 and PS1 in N2A^APP^ cells treated with TDE or scramble peptide (20 μM for 24 h) (*n* = 6 in each group). *P < 0.05 and ***P < 0.001, determined by one-way ANOVA. **e** PS1-Flag and ELK1-HA or p-ELK1-HA, were transfected to HEK293 cells. Coimmunoprecipitation (Co-IP) of exogenous PS1 and ELK1 or p-ELK1 was performed as described above (*n* = 3 in each group). **f** The HEK293 cells were transfected with PS1-Flag and Ub-HA, along with or without ELK1 or p-ELK1, followed by treatment with or without TDE. PS1 ubiquitination was evaluated by Co-IP as described above (*n* = 3 in each group). **g** The HEK293 cells were transfected with PS1-Flag, Ub-HA and SYVN1, along with or without ELK1 or p-ELK1, followed by treatment with or without TDE. PS1 ubiquitination was evaluated by Co-IP as described above (*n* = 3 in each group). ns, not significant.

ELK1 activation is well established to occur through ERK-mediated phosphorylation at Ser383 and Ser389^35,36^. Therefore, we investigated the potential role of phosphorylated ELK1 (p-ELK1) in the SYVN1-mediated ubiquitination and degradation of PS1 in AD. Our findings revealed a significant elevation in p-ELK1 expression in the hippocampus of 6-month-old AD mice (Fig. 5a) and in N2A^APP^ cells (Fig. 5b). In addition, Aβ_1-42_ peptide upregulated p-ELK1 expression in a concentration-dependent manner in N2A cells (Fig. 5c). To test whether p-ELK1 affects PS1 levels, N2A^APP^ cells were treated with ELK1 phosphorylation inhibitor TAT-DEF-ELK1 (TDE) peptide or its scrambled variant (Fig. 5d). Treatment with TDE resulted in a significant reduction in PS1 expression compared with the scramble control (Fig. 5d). To explore the effect of ELK1 phosphorylation on its interaction with PS1, the HEK293 cells were cotransfected with PS1-Flag and either ELK1-HA or HA-tagged p-ELK1 (p-ELK1-HA). The results showed that the interaction between p-ELK1 and PS1 was much stronger than that between ELK1 and PS1 (Fig. 5e). Further experiments aimed at elucidating p-ELK1’s effect on PS1 ubiquitination involved cotransfecting HEK293 cells with PS1-Flag and Ub-HA, alongside either ELK1 or p-ELK1, followed by treatment with TDE or its scramble (Supplementary Fig. 8a, b). The cells expressing p-ELK1 showed a significant reduction in ubiquitinated PS1 levels relative to those expressing ELK1 (Fig. 5f). By contrast, the inhibition of ELK1 phosphorylation by TDE significantly increased the levels of ubiquitinated PS1 (Fig. 5f). To further explore the potential inhibitory effect of p-ELK1 on SYVN1-mediated ubiquitination of PS1, the HEK293 cells were cotransfected with PS1-Flag, Ub-HA and SYVN1 plasmids alongside either ELK1 or p-ELK1 plasmid, followed by TDE or scramble treatment (Supplementary Fig. 8c, d). Consistent with the previous results (Fig. 3d, i), SYVN1 overexpression significantly elevated ubiquitinated PS1 levels, while ELK1 overexpression inhibited this effect (Fig. 5g). Notably, the overexpression of p-ELK1 further decreased the levels of ubiquitinated PS1 compared with overexpression of ELK1 (Fig. 5g). Conversely, the inhibition of ELK1 phosphorylation by TDE completely restored the levels of ubiquitinated PS1 (Fig. 5g). Taken together, these results suggest that ELK1, especially its phosphorylated form, is critical for the SYVN1-mediated ubiquitination and subsequent degradation of PS1.

### Inhibition of ELK1 phosphorylation ameliorates synaptic and cognitive impairments in APP23/PS45 mice

**Fig. 6:**
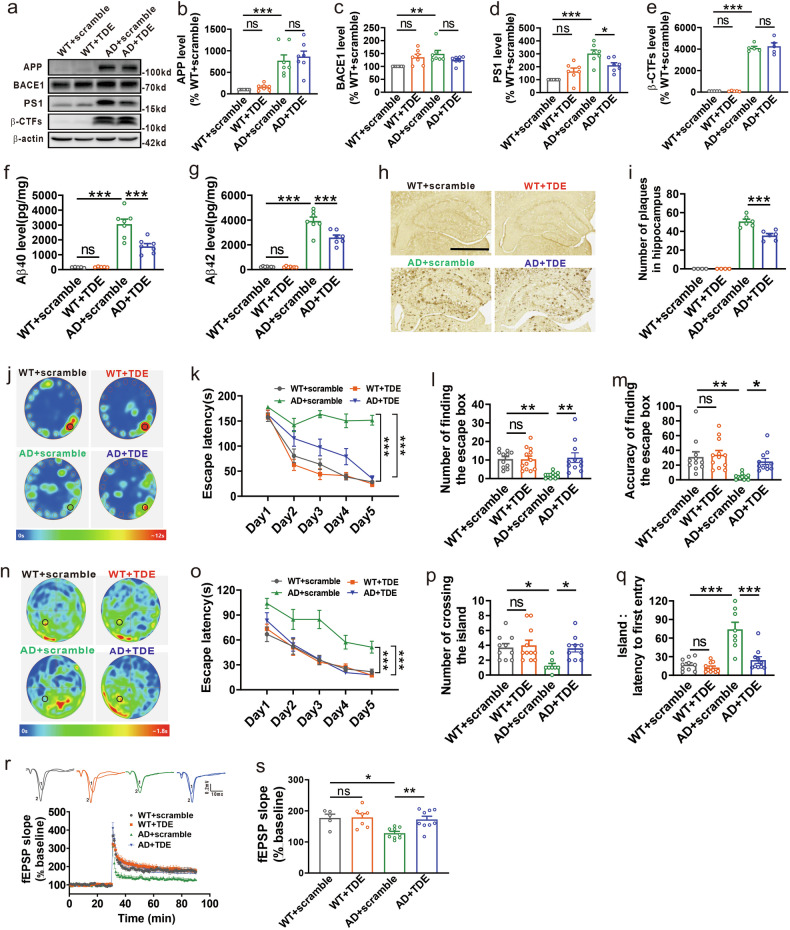
TDE alleviated synaptic and cognitive deficits in APP23/PS45 mice. **a** The mice were subjected to different peptides starting at 2 months: WT mice receiving the scramble peptide (WT + scramble), WT mice receiving TDE (WT + TDE), APP23/PS45 mice receiving the scramble peptide (AD + scramble) and APP23/PS45 mice receiving TDE (AD + TDE). After a month of behavioral experiments which started at the age of 5 months, the mice were subjected to electrophysiological recordings or killed for molecular biological testing. **a**–**e** Western blot of APP (**a**, **b**), BACE1 (**a**, **c**), PS1 (**a**, **d**) and β-CTFs (**a**, **e**) in hippocampus of mice (*n* = 5–7 in each group). *P < 0.05, **P < 0.01 and ***P < 0.001, determined by one-way ANOVA. **f**, **g** Amounts of Aβ_40_ (**f**) and Aβ_42_ (**g**) measured by ELISA in hippocampus of mice (*n* = 5–7 in each group). ***P < 0.001, determined by one-way ANOVA. **h**–**i** Senile plaques detected by immunohistochemistry in hippocampus of mice. Scale bars, 1 mm (*n* = 48–92 slices from four to ten mice in each group). ***P < 0.001, determined by one-way ANOVA. **j** The average heat map during memory retrieval in the Barnes maze test. **k** The latency to locate the escape box during spatial learning in the Barnes maze test (*n* = 11–12 in each group). ***P < 0.001, determined by a repeated measures ANOVA. **l** The number of finding the escape box during memory retrieval (*n* = 11–12 in each group). **P < 0.01, determined by one-way ANOVA. **m** The accuracy of finding the escape box during memory retrieval (*n* = 11–12 in each group). *P < 0.05 and **P < 0.01, determined by a one-way ANOVA. **n** The average heat map during memory retrieval in the Morris maze test. **o** The latency for finding the island during spatial learning in the Morris maze test (*n* = 8–11 in each group). ***P < 0.001, determined by a repeated measures ANOVA. **p** The number of entries into the platform zone during memory retrieval (*n* = 8–11 in each group). *P < 0.05, determined by one-way ANOVA. **q** The latency to first entry to platform area during memory retrieval (*n* = 8–11 in each group). ***P < 0.001, determined by one-way ANOVA. **r** The representative fEPSP traces and normalized slope plots (*n* = 5–9 slices from three to four mice in each group). **s** The bar graphs display the average percentage changes in the fEPSP slope (*n* = 5–9 slices from three to four mice in each group). *P < 0.05 and **P < 0.01, determined by one-way ANOVA. ns, not significiant.

Since TDE-mediated inhibition of ELK1 phosphorylation could promote the ubiquitination and degradation of PS1, we sought to assess whether TDE could alleviate amyloid pathology and cognitive deficits in APP23/PS45 model mice of AD. The mice received intraperitoneal injections of TDE (i.p., 8 mg kg^−1^ per day) starting at 2 months of age, and spatial memory and amyloid pathology were measured at 5 months of age. TDE treatment resulted in a marked decrease in PS1 expression in the hippocampus of AD model mice, without affecting APP, BACE1 or β-CTFs levels (Fig. 6a–e). Notably, TDE treatment had no effect on the expression of all these proteins in WT mice (Fig. 6a–e). As expected, TDE treatment led to substantial decreases in Aβ40 and Aβ42 levels, along with a reduction in senile plaque counts in the hippocampus of AD model mice (Fig. 6f–i).

We also evaluated the effects of TDE on cognitive function in AD model mice by using the Barnes maze and Morris water maze assessments. Compared with the scramble peptide, TDE treatment significantly enhanced spatial learning in AD mice, evidenced by a marked decrease in escape latency during Barnes maze training (Fig. 6k). Furthermore, TDE administration increased the frequency (Fig. 6l) and accuracy (Fig. 6m) of probing escape box during probe test, indicating enhanced memory retrieval by TDE in AD mice. Similar to the results in the Barnes maze test, TDE administration ameliorated spatial learning (Fig. 6o) and memory retrieval (Fig. 6p, q) in the Morris water maze test in AD model mice. Notably, TDE did not affect spatial learning or memory performance in WT mice in both behavioral tests (Fig. 6j–q).

Similarly, we also measured the effects of TDE on hippocampal CA1 LTP. Consistent with our expectations, TDE restored the impaired LTP to control levels in AD model mice (Fig. 6r, s). Overall, these results demonstrate that inhibiting ELK1 phosphorylation with TDE could ameliorate neuropathology and alleviates synaptic and memory impairments in AD mice.

## Discussion

Our recent study found that ERK1/2 is excessively activated in AD, and inhibiting this activation using U0126 effectively lowers the levels of APP and BACE1, subsequently reducing Aβ generation^32^. However, the downstream signals of ERK1/2 involved in APP processing are still unclear. ELK1, a principal target of ERK1/2, undergoes rapid phosphorylation at Ser383 and Ser389 upon ERK1/2 activation. Increasing evidence supports a strong association between elevated ELK1 or p-ELK1 levels and neurodegenerative diseases including AD, and that inhibiting ELK1 may offer neuroprotective functions. For example, T417^+^ELK1, a phosphoform of ELK1, has been found to colocalize with neuritic plaques and neurofibrillary tangles in the brains of patients with AD^42^. Meanwhile, downregulation of ELK1 has been shown to restore Aβ-induced synaptic dysfunction in rat hippocampal neurons^54^. Consistent with these reports, our data reveal a significant increase in ELK1 expression in AD patients and models (Fig. 1). Genetically knocking down ELK1 by shRNA or pharmacologically inhibiting ELK1 by TDE reduces amyloidogenic cleavage of APP, thereby inhibiting senile plaque formation and improving synaptic and cognitive deficits in APP23/PS45 double-transgenic AD model mice (Figs. 4 and 6).

ELK1 primarily functions as a transcription factor, regulating gene expression through its interaction with a serum response factor dimer^55,56^. Meanwhile, our recent report indicates ERK1/2, which acts upstream of ELK1, is implicated in AD pathogenesis via transcriptional regulation of APP and BACE1^32^. Consequently, we hypothesized that the elevated ELK1 expression in AD might also affect the transcription of crucial proteins like APP, BACE1 and PS1, contributing to Aβ production. However, experiments reveal that neither overexpressing nor knocking down ELK1 affects the mRNA levels of APP, BACE1 or PS1 in AD model cells because ELK1 could not bind to their promoters (Supplementary Fig. 3). Notably, these findings contradict previous reports suggesting that ELK1 represses PS1 transcription by specifically interacting with the -10 region of the PS1 promoter in yeast^57,58^. The contradictions between these previous reports and our present results warrant further investigation, potentially arising from differences in species or experimental methodologies employed in those studies.

Nevertheless, we found that knocking down ELK1 effectively reduces APP amyloidogenic processing by decreasing PS1 protein levels, whereas ELK1 overexpression does not alter PS1 levels in AD (Figs. 2 and 4). One possibility is that the already elevated ELK1 levels in AD are sufficient to maintain high PS1 expression, making additional ELK1 overexpression ineffective in further increasing PS1 levels. Since ELK1 does not affect PS1 transcription, it probably regulates PS1 degradation, which is typically modulated by either the ubiquitin–proteasome system or the autophagy–lysosome pathway^48^. Notably, our results indicate that ELK1 is not involved in autophagy-mediated PS1 degradation but rather inhibits PS1 ubiquitination and subsequent degradation (Fig. 2). Ubiquitin contains seven lysine (K) residues (K6, K11, K27, K29, K33, K48 and K63), each capable of forming polyubiquitination chains on substrates. Structural studies have shown that K27 is the least solvent-exposed lysine residue in ubiquitin molecules^59^, suggesting it may be less accessible for enzymatic modification, which could explain its lower abundance in cells. In additon, the enzymes that specifically catalyze K27-linked ubiquitination, including ubiquitin ligases and deubiquitinating enzymes, remain largely uncharacterized. While K48-linked ubiquitination is traditionally associated with proteasomal degradation, the functional role of K27-linked ubiquitination is still under investigation. Studies have implicated K27-linked ubiquitination in DNA damage repair^60^, immune responses^61,62^ and antiviral activity^63,64^. Interestingly, tumor necrosis factor receptor-associated factor 6 (TRAF6)-mediated K27-linked ubiquitination has been shown to modify proteins such as α-synuclein and DJ-1 in Parkinson’s disease and huntingtin in Huntington’s disease^65,66^, suggesting a role in neurodegenerative diseases. Our findings align with these observations, as PS1 is predominantly modified by K27-linked ubiquitination, indicating that this modification may play a role in AD-related mechanisms (Fig. 2). However, the precise function of K27-linked ubiquitination in PS1 degradation remains unclear and warrants further investigation.

Previous studies have identified TRAF6 and SEL-10 as E3 ubiquitin ligases for PS1. TRAF6 mediates K63-linked ubiquitination of PS1, which increases PS1 protein levels without affecting γ-secretase activity or Aβ production^67^. This suggests that TRAF6-mediated ubiquitination does not regulate PS1 degradation. Similarly, while SEL-10 interacts with PS1 and promotes its ubiquitination and degradation, it has also been shown to enhance APP amyloidogenic processing and Aβ generation^68^. This effect may stem from SEL-10 not only ubiquitinating PS1 but also increasing BACE1 activity, thereby promoting Aβ production. Since SEL-10 may not specifically target PS1 in APP metabolism, we sought to identify novel E3 ligases for PS1. Based on predictions from the UbiBrowser E3 ligase database and our experimental results, we propose that SYVN1 functions as an E3 ligase for PS1. Furthermore, ELK1 inhibits SYVN1-mediated PS1 ubiquitination and degradation, leading to increased γ-secretase activity and Aβ production, highlighting the potential regulatory role of SYVN1 in AD pathogenesis (Fig. 3).

As ELK1 has been excluded as a transcription factor influencing PS1 expression and Aβ generation, we propose that post-translational modifications of extranuclear ELK1, particularly phosphorylation, may facilitate Aβ generation by modulating its interactions with PS1. Indeed, we report that p-ELK1 levels are apparently increased in AD, whereas genetically knocking down ELK1 or pharmacologically inhibiting ELK1 phosphorylation enhances SYVN1-mediated ubiquitination and degradation of PS1, resulting in decreased Aβ generation and improved synaptic and cognitive function in AD model mice (Figs. 3–6). These data suggest that strategies targeting ELK1 inhibition, such as TDE, which specifically interferes with DEF docking domain of ELK1 to inhibit its phosphorylation and nuclear translocation^44^, may enhance learning and memory capabilities, potentially offering therapeutic benefits for cognitive disorders such as AD. However, ELK1 plays critical roles in various cellular processes, including proliferation, apoptosis, thymocyte development, glucose homeostasis and brain function^69^. To minimize the potential impact of ELK1 inhibition on normal physiological functions, we monitored several parameters to assess the potential side effects of TDE treatment. Specifically, we measured body weight, mortality rate and conducted behavioral assays, including the open field test and elevated plus maze test, to evaluate the effects of TDE on spontaneous activity and anxiety/depressive-like behaviors. Our results indicated that TDE treatment at currently used doses did not cause any adverse effects on these parameters (Supplementary Fig. 9). Nevertheless, we acknowledge that other potential side effects cannot be entirely ruled out, and further studies are needed to comprehensively assess the long-term safety profile of TDE treatment. Although we here reveal that TDE improves the pathology of AD, it may also inhibit the normal physiological function of ELK1. Therefore, a key avenue for future research will be to pinpoint the ELK1 recognition sites on PS1, facilitating further structural investigations into the ELK1-PS1 interaction and enabling the rational design of small molecule drugs or peptides that can disrupt this interaction without compromising ELK1’s physiological roles.

**Fig. 7:**
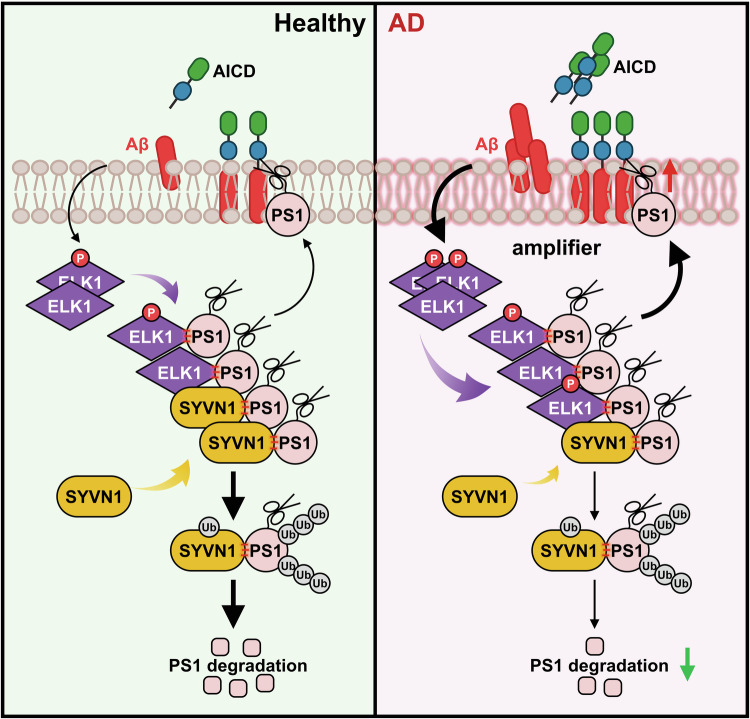
Schematic illustration presumed the mechanism of ELK1 in AD. ELK1, especially its phosphorylated form p-ELK1, is significantly increased in AD, facilitating the amyloidogenic processing of APP. This occurs through the inhibition of SYVN1-mediated ubiquitination and degradation of PS1, ultimately resulting in the accumulation of senile plaques and subsequent deficits in synaptic and cognitive functions.

In summary, our research highlights that ELK1, especially its phosphorylated form p-ELK1, is aberrantly elevated and plays critical roles in the pathogenesis of AD (Fig. 7). Both genetic knockdown and pharmacological inhibition of ELK1 reduce APP amyloidogenic processing by promoting the SYVN1-mediated ubiquitination and degradation of PS1, thereby inhibiting Aβ generation and alleviating synaptic and cognitive impairments in a mouse model of AD. These findings reveal new insights into the involvement of ELK1 in AD pathogenesis and provide scientific basis for the development of ELK1 inhibitors aimed at addressing learning and memory disorders in individuals with AD as well as in the aging population.

## Supplementary Information

Supplementary Information

## Data Availability

The data that support the findings of this study are available from the corresponding author upon reasonable request.

## References

[1] Austad, S. N. et al. Targeting whole body metabolism and mitochondrial bioenergetics in the drug development for Alzheimer’s disease. *Acta Pharmaceutica Sin. B* **12**, 511–531 (2022). 10.1016/j.apsb.2021.06.014 PMC8897048 35256932

[2] Alzheimer’s Association. Alzheimer’s disease facts and figures. *Alzheimer’s Dement.* **20**, 3708–3821 (2024). 10.1002/alz.13809 PMC11095490 38689398

[3] Bloom, G. S. Amyloid-beta and tau: the trigger and bullet in Alzheimer disease pathogenesis. *JAMA Neurol.* **71**, 505–508 (2014). 24493463 10.1001/jamaneurol.2013.5847 PMC12908160

[4] Li, Y. M. et al. Presenilin 1 is linked with gamma-secretase activity in the detergent solubilized state. *Proc. Natl Acad. Sci. USA* **97**, 6138–6143 (2000). 10801983 10.1073/pnas.110126897 PMC18571

[5] Bossy-Wetzel, E., Schwarzenbacher, R. & Lipton, S. A. Molecular pathways to neurodegeneration. *Nat. Med.* **10**, S2–S9 (2004). 15272266 10.1038/nm1067

[6] He, C. et al. Epigenetic regulation of amyloid-beta metabolism in Alzheimer’s disease. *Curr. Med. Sci.* **40**, 1022–1030 (2021). 10.1007/s11596-020-2283-0 33428129

[7] Estus, S. et al. Potentially amyloidogenic, carboxyl-terminal derivatives of the amyloid protein precursor. *Science* **255**, 726–728 (1992). 1738846 10.1126/science.1738846

[8] Golde, T. E., Estus, S., Younkin, L. H., Selkoe, D. J. & Younkin, S. G. Processing of the amyloid protein precursor to potentially amyloidogenic derivatives. *Science* **255**, 728–730 (1992). 1738847 10.1126/science.1738847

[9] Vassar, R. et al. Beta-secretase cleavage of Alzheimer’s amyloid precursor protein by the transmembrane aspartic protease BACE. *Science* **286**, 735–741 (1999). 10531052 10.1126/science.286.5440.735

[10] Xuefei, G. et al. Molecular mechanism of substrate recognition and cleavage by human γ-secretase. *Science* **384**, 1091–1095 (2024). 38843321 10.1126/science.adn5820

[11] Zhou, R. et al. Recognition of the amyloid precursor protein by human gamma-secretase. *Science* **363**, eaaw0930 (2019). 30630874 10.1126/science.aaw0930

[12] De Strooper, B. Aph-1, Pen-2, and nicastrin with presenilin generate an active gamma-secretase complex. *Neuron* **38**, 9–12 (2003). 12691659 10.1016/s0896-6273(03)00205-8

[13] De Strooper, B. & Karran, E. New precision medicine avenues to the prevention of Alzheimer’s disease from insights into the structure and function of γ-secretases. *EMBO J.* **43**, 887–903 (2024). 38396302 10.1038/s44318-024-00057-w PMC10943082

[14] Samuels, I. S. et al. Deletion of ERK2 mitogen-activated protein kinase identifies its key roles in cortical neurogenesis and cognitive function. *J. Neurosci.* **28**, 6983–6995 (2008). 18596172 10.1523/JNEUROSCI.0679-08.2008 PMC4364995

[15] Samuels, I. S., Saitta, S. C. & Landreth, G. E. MAP’ing CNS development and cognition: an ERKsome process. *Neuron* **61**, 160–167 (2009). 19186160 10.1016/j.neuron.2009.01.001 PMC3663441

[16] Li, X. et al. MEK Is a key regulator of gliogenesis in the developing brain. *Neuron* **75**, 1035–1050 (2012). 22998872 10.1016/j.neuron.2012.08.031 PMC3483643

[17] Di Cristo, G. et al. Requirement of ERK activation for visual cortical plasticity. *Science* **292**, 2337–2340 (2001). 11423664 10.1126/science.1059075

[18] Thiels, E., Kanterewicz, B. I., Norman, E. D., Trzaskos, J. M. & Klann, E. Long-term depression in the adult hippocampus in vivo involves activation of extracellular signal-regulated kinase and phosphorylation of Elk-1. *J. Neurosci.* **22**, 2054–2062 (2002). 11896145 10.1523/JNEUROSCI.22-06-02054.2002 PMC6758273

[19] Impey, S., Obrietan, K. & Storm, D. R. Making new connections: role of ERK/MAP kinase signaling in neuronal plasticity. *Neuron* **23**, 11–14 (1999). 10402188 10.1016/s0896-6273(00)80747-3

[20] Kanterewicz, B. I. et al. The extracellular signal-regulated kinase cascade is required for NMDA receptor-independent LTP in area CA1 but not area CA3 of the hippocampus. *J. Neurosci.* **20**, 3057–3066 (2000). 10777769 10.1523/JNEUROSCI.20-09-03057.2000 PMC6773121

[21] Schafe, G. E. et al. Activation of ERK/MAP kinase in the amygdala is required for memory consolidation of pavlovian fear conditioning. *J. Neurosci.* **20**, 8177–8187 (2000). 11050141 10.1523/JNEUROSCI.20-21-08177.2000 PMC6772720

[22] Atkins, C. M., Selcher, J. C., Petraitis, J. J., Trzaskos, J. M. & Sweatt, J. D. The MAPK cascade is required for mammalian associative learning. *Nat. Neurosci.* **1**, 602–609 (1998). 10196568 10.1038/2836

[23] Feld, M., Dimant, B., Delorenzi, A., Coso, O. & Romano, A. Phosphorylation of extra-nuclear ERK/MAPK is required for long-term memory consolidation in the crab. *Chasmagnathus. Behav. Brain Res.* **158**, 251–261 (2005). 15698891 10.1016/j.bbr.2004.09.005

[24] Igaz, L. M. et al. Early activation of extracellular signal-regulated kinase signaling pathway in the hippocampus is required for short-term memory formation of a fear-motivated learning. *Cell Mol. Neurobiol.* **26**, 989–1002 (2006). 16977492 10.1007/s10571-006-9116-y PMC11520636

[25] Kelly, A., Laroche, S. & Davis, S. Activation of mitogen-activated protein kinase/extracellular signal-regulated kinase in hippocampal circuitry is required for consolidation and reconsolidation of recognition memory. *J. Neurosci.* **23**, 5354–5360 (2003). 12832561 10.1523/JNEUROSCI.23-12-05354.2003 PMC6741214

[26] Villarreal, J. S. & Barea-Rodriguez, E. J. ERK phosphorylation is required for retention of trace fear memory. *Neurobiol. Learn Mem.* **85**, 44–57 (2006). 16182574 10.1016/j.nlm.2005.08.005

[27] Satoh, Y. et al. Extracellular signal-regulated kinase 2 (ERK2) knockdown mice show deficits in long-term memory; ERK2 has a specific function in learning and memory. *J. Neurosci.* **27**, 10765–10776 (2007). 17913910 10.1523/JNEUROSCI.0117-07.2007 PMC6672813

[28] Ferrer, I. et al. Phosphorylated map kinase (ERK1, ERK2) expression is associated with early tau deposition in neurones and glial cells, but not with increased nuclear DNA vulnerability and cell death, in Alzheimer disease, Pick’s disease, progressive supranuclear palsy and corticobasal degeneration. *Brain Pathol.* **11**, 144–158 (2001). 11303790 10.1111/j.1750-3639.2001.tb00387.x PMC8098611

[29] Pei, J. J. et al. Up-regulation of mitogen-activated protein kinases ERK1/2 and MEK1/2 is associated with the progression of neurofibrillary degeneration in Alzheimer’s disease. *Brain Res. Mol. Brain Res.* **109**, 45–55 (2002). 12531514 10.1016/s0169-328x(02)00488-6

[30] Siano, G. et al. Identification of an ERK inhibitor as a therapeutic drug against tau aggregation in a new cell-based assay. *Front. Cell Neurosci.* **13**, 386 (2019). 31496937 10.3389/fncel.2019.00386 PMC6712076

[31] Jin, P., Choi, D. Y. & Hong, J. T. Inhibition of extracellular signal-regulated kinase activity improves cognitive function in Tg2576 mice. *Clin. Exp. Pharm. Physiol.* **39**, 852–857 (2012). 10.1111/j.1440-1681.2012.12000.x 23013130

[32] Du, Y. et al. MKP-1 reduces Abeta generation and alleviates cognitive impairments in Alzheimer’s disease models. *Signal Transduct. Target Ther.* **4**, 58 (2019). 31840000 10.1038/s41392-019-0091-4 PMC6895219

[33] Luo, M. et al. miR-429-3p mediates memory decline by targeting MKP-1 to reduce surface GluA1-containing AMPA receptors in a mouse model of Alzheimer’s disease. *Acta Pharmaceutica Sin. B* **14**, 635–652 (2024). 10.1016/j.apsb.2023.10.015 PMC10840427 38322333

[34] Kasza, A. Signal-dependent Elk-1 target genes involved in transcript processing and cell migration. *Biochim Biophys. Acta* **1829**, 1026–1033 (2013). 23711433 10.1016/j.bbagrm.2013.05.004

[35] Sun, J. & Nan, G. The extracellular signal-regulated kinase 1/2 pathway in neurological diseases: a potential therapeutic target (Review). *Int J. Mol. Med.* **39**, 1338–1346 (2017). 28440493 10.3892/ijmm.2017.2962 PMC5428947

[36] Chuderland, D. & Seger, R. Protein–protein interactions in the regulation of the extracellular signal-regulated kinase. *Mol. Biotechnol.* **29**, 57–74 (2005). 15668520 10.1385/MB:29:1:57

[37] Li, P. et al. ELK1-mediated YTHDF1 drives prostate cancer progression by facilitating the translation of Polo-like kinase 1 in an m6A dependent manner. *Int. J. Biol. Sci.* **18**, 6145–6162 (2022). 36439881 10.7150/ijbs.75063 PMC9682537

[38] Sgambato, V. et al. In vivo expression and regulation of Elk-1, a target of the extracellular-regulated kinase signaling pathway, in the adult rat brain. *J. Neurosci.* **18**, 214–226 (1998). 9412502 10.1523/JNEUROSCI.18-01-00214.1998 PMC6793414

[39] Barrett, L. E. et al. Region-directed phototransfection reveals the functional significance of a dendritically synthesized transcription factor. *Nat. Methods* **3**, 455–460 (2006). 16721379 10.1038/nmeth885

[40] Barrett, L. E. et al. Elk-1 associates with the mitochondrial permeability transition pore complex in neurons. *Proc. Natl Acad. Sci. USA* **103**, 5155–5160 (2006). 16549787 10.1073/pnas.0510477103 PMC1458810

[41] Lu, Z., Miao, Z., Zhu, J. & Zhu, G. ETS-domain containing protein (Elk1) suppression protects cortical neurons against oxygen-glucose deprivation injury. *Exp. Cell Res.* **371**, 42–49 (2018). 30053446 10.1016/j.yexcr.2018.07.038

[42] Sharma, A. et al. A neurotoxic phosphoform of Elk-1 associates with inclusions from multiple neurodegenerative diseases. *PLoS ONE* **5**, e9002 (2010). 20126313 10.1371/journal.pone.0009002 PMC2814869

[43] Lai, Y. J. et al. Estrogen receptor alpha promotes Cav1.2 ubiquitination and degradation in neuronal cells and in APP/PS1 mice. *Aging Cell* **18**, e12961 (2019). 31012223 10.1111/acel.12961 PMC6612642

[44] Lavaur, J. et al. A TAT-DEF-Elk-1 peptide regulates the cytonuclear trafficking of Elk-1 and controls cytoskeleton dynamics. *J. Neurosci.* **27**, 14448–14458 (2007). 18160653 10.1523/JNEUROSCI.2279-07.2007 PMC6673434

[45] Dong, Z. et al. Long-term potentiation decay and memory loss are mediated by AMPAR endocytosis. *J. Clin. Invest.* **125**, 234–247 (2015). 25437879 10.1172/JCI77888 PMC4382266

[46] Thinakaran, G. et al. Endoproteolysis of presenilin 1 and accumulation of processed derivatives in vivo. *Neuron* **17**, 181–190 (1996). 8755489 10.1016/s0896-6273(00)80291-3

[47] Li, Y. et al. Structural biology of presenilin 1 complexes. *Mol. Neurodegener.* **9**, 59 (2014). 25523933 10.1186/1750-1326-9-59 PMC4326451

[48] Sun-Wang, J. L., Ivanova, S. & Zorzano, A. The dialogue between the ubiquitin–proteasome system and autophagy: Implications in ageing. *Ageing Res. Rev.* **64**, 101203 (2020). 33130248 10.1016/j.arr.2020.101203

[49] Duggan, S. P., Yan, R. & McCarthy, J. V. A ubiquitin-binding CUE domain in presenilin-1 enables interaction with K63-linked polyubiquitin chains. *FEBS Lett.* **589**, 1001–1008 (2015). 25796185 10.1016/j.febslet.2015.03.008

[50] Fujita, H. et al. The E3 ligase synoviolin controls body weight and mitochondrial biogenesis through negative regulation of PGC-1beta. *EMBO J.* **34**, 1042–1055 (2015). 25698262 10.15252/embj.201489897 PMC4406651

[51] Yagishita, N. et al. RING-finger type E3 ubiquitin ligase inhibitors as novel candidates for the treatment of rheumatoid arthritis. *Int J. Mol. Med.* **30**, 1281–1286 (2012). 22992760 10.3892/ijmm.2012.1129 PMC4042867

[52] Wu, T. et al. Hrd1 suppresses Nrf2-mediated cellular protection during liver cirrhosis. *Genes Dev.* **28**, 708–722 (2014). 24636985 10.1101/gad.238246.114 PMC4015486

[53] Bianchini, E., Fanin, M., Mamchaoui, K., Betto, R. & Sandona, D. Unveiling the degradative route of the V247M alpha-sarcoglycan mutant responsible for LGMD-2D. *Hum. Mol. Genet.* **23**, 3746–3758 (2014). 24565866 10.1093/hmg/ddu088 PMC4065151

[54] Szatmari, E. M., Oliveira, A. F., Sumner, E. J. & Yasuda, R. Centaurin-alpha1-Ras-Elk-1 signaling at mitochondria mediates beta-amyloid-induced synaptic dysfunction. *J. Neurosci.* **33**, 5367–5374 (2013). 23516302 10.1523/JNEUROSCI.2641-12.2013 PMC3866502

[55] Herrera, R. E., Shaw, P. E. & Nordheim, A. Occupation of the c-fos serum response element in vivo by a multi-protein complex is unaltered by growth factor induction. *Nature* **340**, 68–70 (1989). 2786995 10.1038/340068a0

[56] Shaw, P. E., Schroter, H. & Nordheim, A. The ability of a ternary complex to form over the serum response element correlates with serum inducibility of the human c-*fos*promoter. *Cell* **56**, 563–572 (1989). 2492906 10.1016/0092-8674(89)90579-5

[57] Pastorcic, M. & Das, H. K. Ets transcription factors ER81 and Elk1 regulate the transcription of the human presenilin 1 gene promoter. *Brain Res. Mol. Brain Res.* **113**, 57–66 (2003). 12750007 10.1016/s0169-328x(03)00090-1

[58] Pastorcic, M. & Das, H. K. An abbreviated procedure for the cloning and identification of Ets transcription factors regulating the expression of the human presenilin 1 gene. *Brain Res. Brain Res. Protoc.* **12**, 35–40 (2003). 12928043 10.1016/s1385-299x(03)00069-2

[59] Castaneda, C. A. et al. Linkage via K27 bestows ubiquitin chains with unique properties among polyubiquitins. *Structure* **24**, 423–436 (2016). 26876099 10.1016/j.str.2016.01.007 PMC4787624

[60] Gatti, M. et al. RNF168 promotes noncanonical K27 ubiquitination to signal DNA damage. *Cell Rep.* **10**, 226–238 (2015). 25578731 10.1016/j.celrep.2014.12.021

[61] Xue B. et al. TRIM21 promotes innate immune response to RNA viral infection through Lys27-linked polyubiquitination of MAVS. *J Virol*. **92**, e00321–18 (2018). 10.1128/JVI.00321-18 PMC6026736 29743353

[62] Liu, J. et al. Rhbdd3 controls autoimmunity by suppressing the production of IL-6 by dendritic cells via K27-linked ubiquitination of the regulator NEMO. *Nat. Immunol.* **15**, 612–622 (2014). 24859449 10.1038/ni.2898

[63] Zhang Y. et al. Porcine RING finger protein 114 inhibits classical swine fever virus replication via K27-linked polyubiquitination of viral NS4B. *J Virol*. **93**, e01741–18 (2019). 10.1128/JVI.01248-19 PMC6803260 31413123

[64] Li, Z. et al. Zebrafish F-box protein fbxo3 negatively regulates antiviral response through promoting K27-linked polyubiquitination of the transcription factors irf3 and irf7. *J. Immunol.* **205**, 1897–1908 (2020). 32859728 10.4049/jimmunol.2000305

[65] Zucchelli, S. et al. TRAF6 promotes atypical ubiquitination of mutant DJ-1 and alpha-synuclein and is localized to Lewy bodies in sporadic Parkinson’s disease brains. *Hum. Mol. Genet* **19**, 3759–3770 (2010). 20634198 10.1093/hmg/ddq290

[66] Zucchelli, S. et al. Tumor necrosis factor receptor-associated factor 6 (TRAF6) associates with huntingtin protein and promotes its atypical ubiquitination to enhance aggregate formation. *J. Biol. Chem.* **286**, 25108–25117 (2011). 21454471 10.1074/jbc.M110.187591 PMC3137084

[67] Yan, R., Farrelly, S. & McCarthy, J. V. Presenilins are novel substrates for TRAF6-mediated ubiquitination. *Cell Signal* **25**, 1769–1779 (2013). 23707529 10.1016/j.cellsig.2013.05.015

[68] Li, J. et al. SEL-10 interacts with presenilin 1, facilitates its ubiquitination, and alters A-beta peptide production. *J. Neurochem* **82**, 1540–1548 (2002). 12354302 10.1046/j.1471-4159.2002.01105.x

[69] Thiel, G., Backes, T. M., Guethlein, L. A. & Rossler, O. G. Critical protein–protein interactions determine the biological activity of Elk-1, a master regulator of stimulus-induced gene transcription. *Molecules* **26**, 6125 (2021). 34684708 10.3390/molecules26206125 PMC8541449

