# Supplementary material for: ELK1 inhibition alleviates amyloid pathology and memory decline by promoting the SYVN1-mediated ubiquitination and degradation of PS1 in Alzheimer’s disease
Source: Exp Mol Med. 2025 May 1;57(5):1032–46. doi: 10.1038/s12276-025-01455-8 (PMC12130275; doi:10.1038/s12276-025-01455-8)
Supplement: Supplementary file 1 — Supplementary Information [file 12276_2025_1455_MOESM1_ESM.pdf]

Supplementary Materials

Supplementary methods

Quantitative real time PCR (qRT-PCR)

Total RNA was extracted from cultured cells using High Pure Total RNA Extraction Kit (Bio Teke, Peking, China) according to the manufacturer's instructions. Briefly, the RNA concentration and purity were determined using a spectrophotometer NanoDrop 2000 (Nanodrop Technologies, Wilmington, DE, USA). Subsequently, 1μg of RNA was reverse-transcribed into cDNA using the PrimeScript™ RT Reagent Kit (Takara, Otsu, Shiga, Japan). For quantitative real-time PCR (qRT-PCR), SYBR Premix Ex Taq II (Takara, Otsu, Shiga, Japan) was used to perform on a CFX Manager software (Bio-Rad). The primer sequences used in our study were as follows: APP (forward: 5′-TTGTAAGTGATGCCCTTCTCGTT, reverse: 5′-AGCAACATGCCGTAGTCATGCAA); BACE1 (forward: 5′-CAGCTCCTTAAACTGACGCTA, reverse: 5′-TCCTTTCTGCCTTTGATACTCT); PS1 (forward: 5′-CATATTTGCGGTTAGAATCCCA, reverse: 5′-CAAAGTCCAATAGTGCAAGGT); β-actin (forward: 5′-CCTTCCTGGGCATGGAGTC, reverse: 5′-TGATCTTCATTGTGCTGGGTG). β-actin was utilized as an internal control for normalization, and the relative expression levels of APP, BACE1, and PS1 were normalized to β-actin levels.

Electrophoretic mobility shift assay (EMSA)

The EMSA was conducted as previously described, and the sequences of the wild type (WT) and mutated (Mut) oligonucleotides used are provided in Supplementary Table 3. Briefly, nuclear proteins from HEK293 cells that were transfected with a plasmid overexpressing ELK1 were extracted using the Minute™ Cytoplasmic and Nuclear Fractionation Kit (Invent Biotechnologies, EdenPrairie, USA). The protein concentration was determined using the BCA Protein Assay Kit, and 2 μg of nuclear protein was incubated with an Alexa Fluor 700-labeled ELK1 oligonucleotide (5’-GGATGTCCATATTAGGACATCT) probe at room temperature for 30 minutes in the dark. In the competition assay, unlabeled wild type and mutant (5’-ATCGCTCCATATTAATCGCTCT) ELK1 oligonucleotides at a 10 to 100-fold molar excess were used to compete for binding. The samples were separated using 4% SDS-PAGE and subsequently scanned by the Odyssey system (LI-COR Biosciences, Lincoln, USA).

Elevated plus maze test

The elevated plus maze test was used to assess the anxiety/depressive-like behaviors of the experimental animals. The maze consisted of a cross-shaped structure elevated 60 cm above the ground, with each arm measuring about 80 cm in length. A CCD camera was suspended above the maze to capture the animal's tracking data, which was analyzed using the ANY-maze video tracking system (Stoelting, USA). During the test, mice were placed in the center of the maze and allowed to explore freely for 5 minutes. Anxiety/depressive-like behavior was evaluated by analyzing the percentage of time spent in the open arms and the percentage of entries into the open arms.

Open field test

The open field test was used to assess the spontaneous activity and anxiety/depressive-like behaviors of the experimental animals. The apparatus consisted of an open field chamber measuring 40×40×60 cm, with a CCD camera suspended above the chamber to capture the animal's tracking data, which was analyzed using the ANY-maze video tracking system. During the test, mice were placed in the center of the open field chamber and allowed to explore freely for 10 minutes. Spontaneous activity was assessed by analyzing the total distance traveled in the chamber, while anxiety/depressive-like behavior was evaluated by analyzing the time spent in center zone and the number of entries into the center zone.

Supplementary Figures

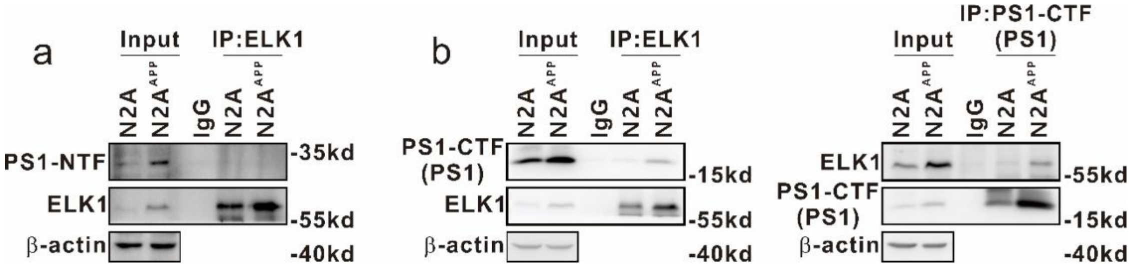

Supplementary Fig. 1: ELK1 interacted specifically with PS1-CTF but not PS1-NTF. (a) Co-IP of endogenous ELK1 and PS1-NTF in N2A and N2A^{APP} cells was performed using antibodies to ELK1. n=4 in each group. **(b)** Co-IP of endogenous ELK1 and PS1-CTF in N2A and N2A^{APP} cells was performed using antibodies to ELK1 and PS1-CTF. n=2-4 in each group.

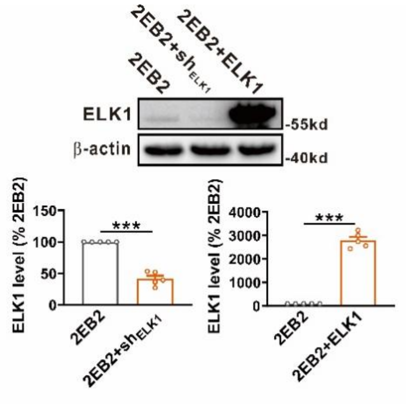

Supplementary Fig. 2: The effectiveness of ELK1 interference and overexpression in 2EB2 cells. ELK1 or sh_{ELK1} plasmid was transfected to 2EB2 cells. ELK1 was determined by WB 48 hours later. n=5 in each group. ***$p<0.001$ by unpaired Student’s t-test.

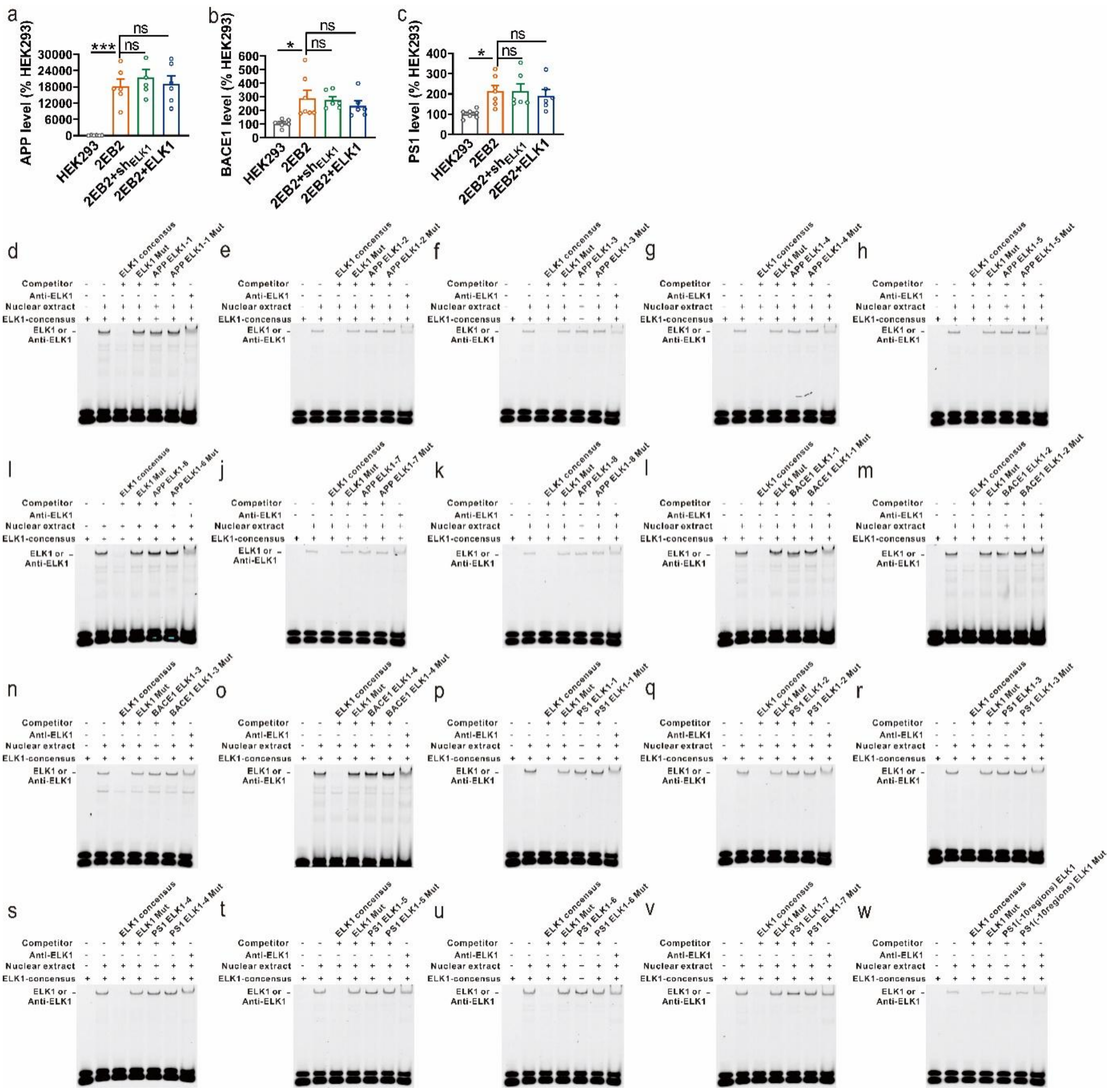

Supplementary Fig. 3: ELK1 did not directly regulate the transcription of APP, BACE1, and PS1. (a-c) ELK1 had no effect on the mRNA levels of APP, BACE1, and PS1. ELK1 or sh_{ELK1} plasmid was transfected to 2EB2 cells. The mRNA levels of APP (a), BACE1 (b), and PS1 (c) were determined by qRT-PCR 48 hours later. *p<0.05 and ***p<0.001 by one-way ANOVA. n=5-7 in each group. **(d-w)** ELK1 did not bind to promoters of APP, BACE1, and PS1 genes. EMSA with APP (d-k), BACE1 (l-o) or PS1 (p-w) and ELK1 probe in nuclear extract of HEK293 cells transfected with plasmid overexpressing ELK1. Lane 1 is the labeled consensus ELK1 probe only. Lane 2 shows a shifted DNA–protein complex formed between the labeled ELK1 and nuclear extracts. Competition assays were performed by further adding

different competitions of oligonucleotides that included consensus wild-type ELK1 (lane 3), mutant ELK1 (lane 4), putative ELK1-binding site in APP, BACE1, PS1 promotor and mutant ELK1-binding site in APP, BACE1, PS1 promotor (lanes 5 and 6). Lane 7 shows the supershifted band with the anti-ELK1 antibody. n=1-3 in each group.

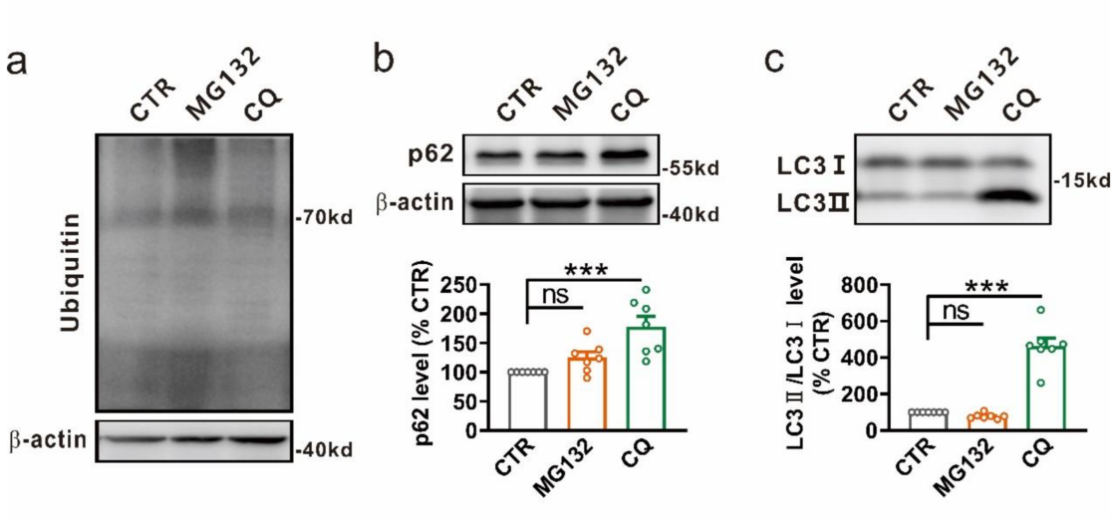

Supplementary Fig. 4: The effectiveness of MG132 and CQ. (a-c) N2A^{APP} cells were exposed to MG132 (10 μM) or chloroquine (CQ, 50 μM). Ubiquitin (a), p62 (b), and LC3 (c) were determined by WB 24 hours later. n=7-8 in each group. ***p<0.001 by one-way ANOVA.

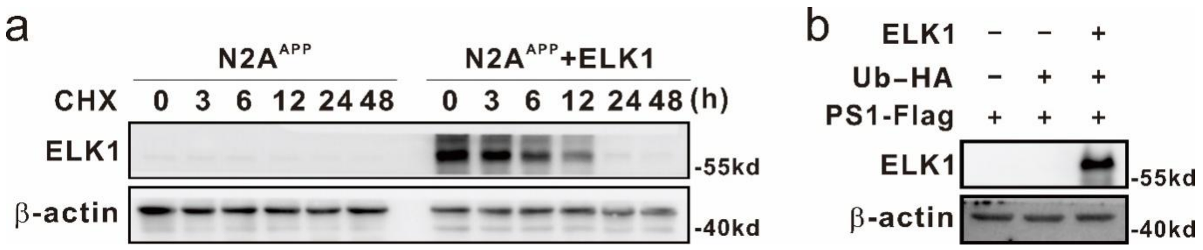

Supplementary Fig. 5: The effectiveness of ELK1 overexpression. (a) N2A^{APP} cells were transfected with or without ELK1, followed by exposure to cycloheximide (CHX, 100 μg/ml) for the indicated time. ELK1 was determined by WB to evaluate its transfection efficiency. n=8 in each group. **(b)** PS1-Flag and Ub-HA, along with or without ELK1, were transfected to HEK293 cells. ELK1 was determined by WB to evaluate its transfection efficiency. n=3 in each group.

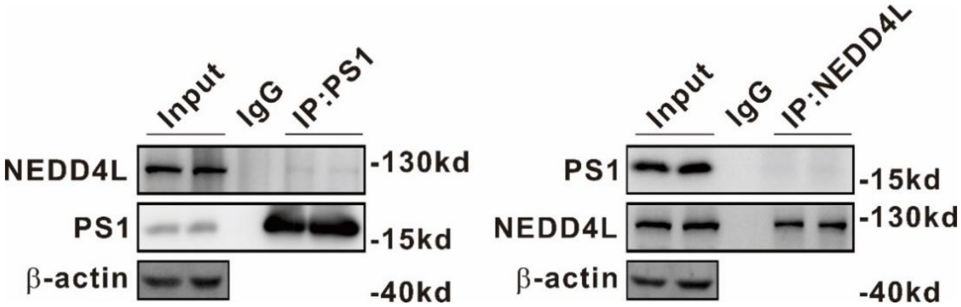

Supplementary Fig. 6: ELK1 did not interact with NEDD4L. Co-IP of endogenous PS1 and NEDD4L in N2A^{APP} cells was performed using antibodies to PS1 and NEDD4L. n=4 in each group.

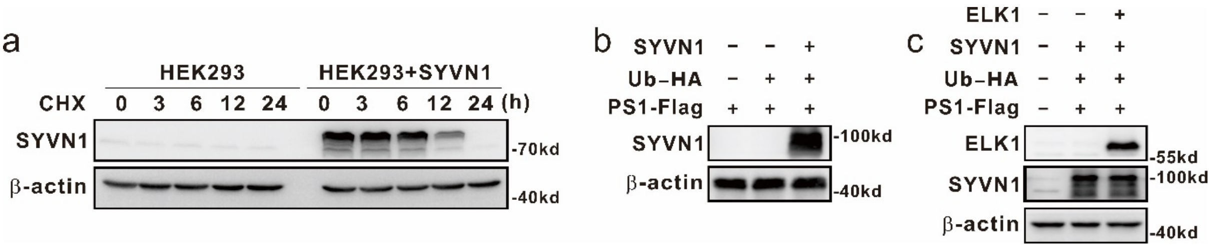

Supplementary Fig. 7: The effectiveness of SYVN1 and ELK1 overexpression. (a) HEK293 cells were transfected with or without SYVN1, followed by exposure to cycloheximide (CHX, 100 μg/ml) for the indicated time. SYVN1 was determined by WB to evaluate its transfection efficiency. n=4 in each group. **(b)** PS1-Flag and Ub-HA, along with or without SYVN1, were transfected to HEK293 cells. SYVN1 was determined by WB to evaluate its transfection efficiency. n=3 in each group. **(c)** PS1-Flag, Ub-HA, and SYVN1, along with or without ELK1, were transfected to HEK293 cells. ELK1 and SYVN1 was determined by WB to evaluate their transfection efficiency. n=3 in each group.

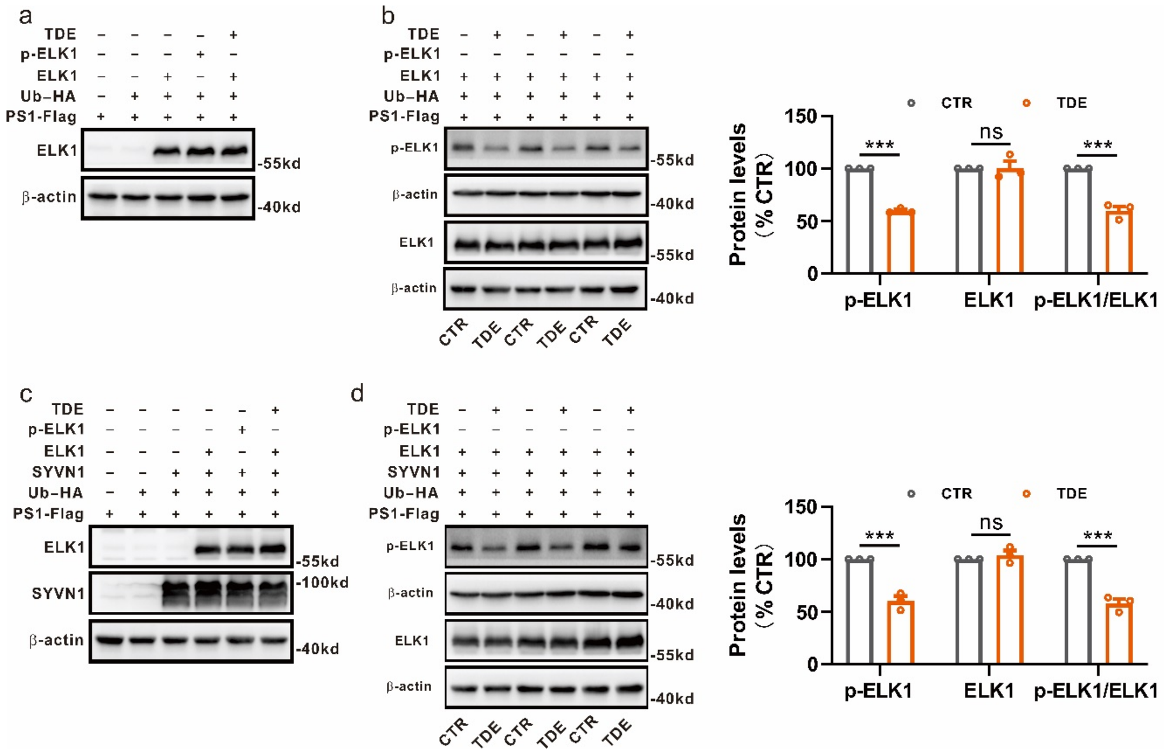

Supplementary Fig. 8: The effectiveness of ELK1 and SYVN1 overexpression, as well as TDE treatment. (a-b) HEK293 cells were transfected with PS1-Flag and Ub-HA, along with or without ELK1 or p-ELK1, followed by treatment with or without TDE. ELK1 was determined by WB to evaluate its transfection efficiency(a). p-ELK1 and ELK1 were determined only in samples transfected with PS1-Flag, Ub-HA and ELK1 along with treatment with or without TDE by WB to evaluate the effectiveness of TDE in inhibiting ELK1 phosphorylation (b). n=3 in each group. **(c-d)** HEK293 cells were transfected with PS1-Flag, Ub-HA, and SYVN1, along with or without ELK1 or p-ELK1, followed by treatment with or without TDE. SYVN1 and ELK1 were determined by WB to evaluate their transfection efficiency (c). p-ELK1 and ELK1 were determined only in samples transfected with PS1-Flag, Ub-HA, SYVN1 and ELK1 along with treatment with or without TDE by WB to evaluate the effectiveness of TDE in inhibiting ELK1 phosphorylation (d). n=3 in each group.

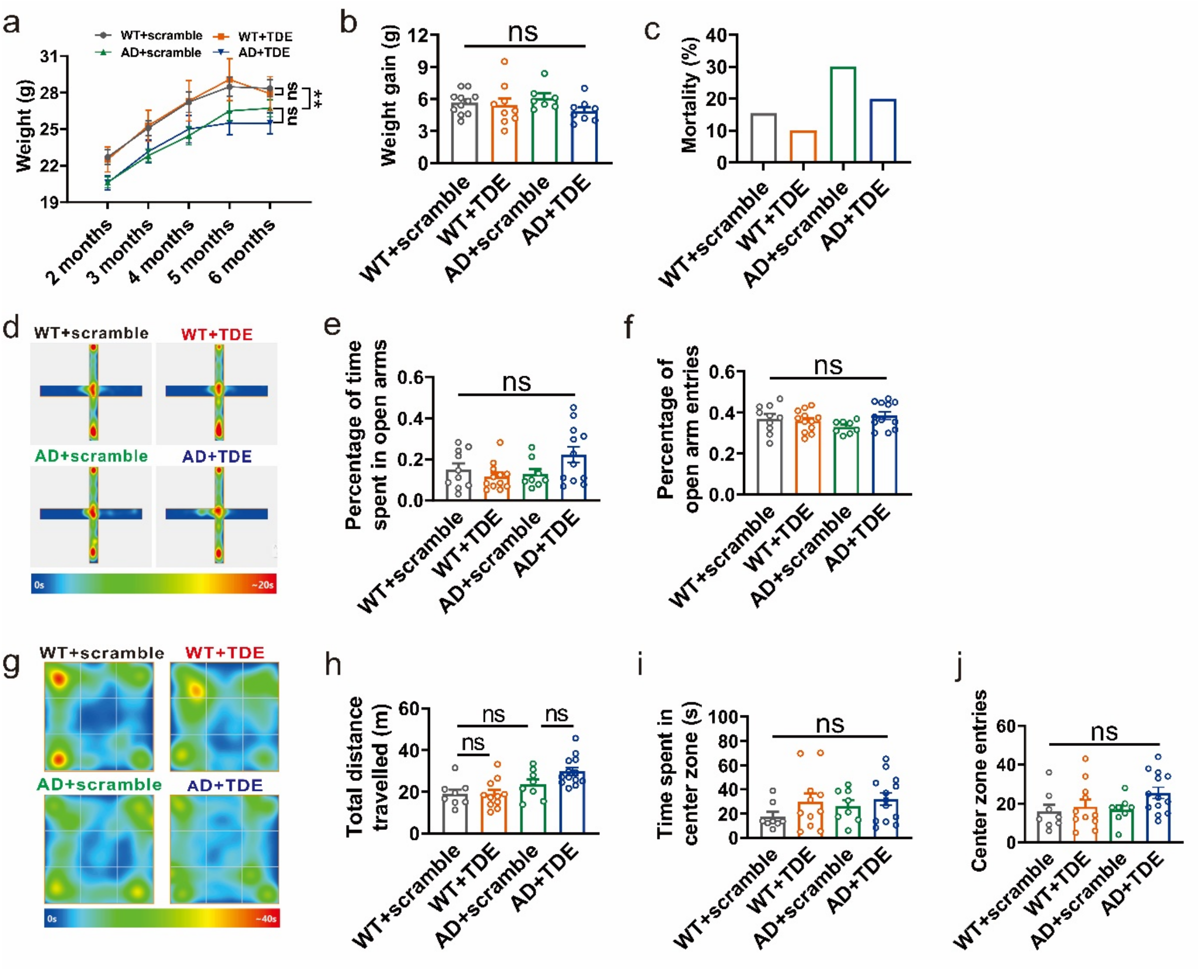

Supplementary Fig. 9:TDE treatment had no effect on the body weight gain, mortality, spontaneous activity, and anxiety/depressive-like behaviors in mice. (a-j) Mice were subjected to different peptides starting at 2 months: WT mice received the scramble peptide (WT + scramble), WT mice received TDE (WT + TDE), APP23/PS45 mice received the scramble peptide (AD + scramble), and APP23/PS45 mice received TDE (AD + TDE). After a month of behavioral experiments which started at the age of 5 months, mice were subjected to electrophysiological recordings or sacrificed for molecular biological testing. **(a)** Body weight of mice during peptide treatment. n=7-11 in each group. **p<0.01 by repeated measures ANOVA. **(b)** Body weight gain of mice before and after peptide treatment. n=7-11 in each group. **(c)** Mortality of mice during peptide treatment. **(d)** Average heatmap in elevated plus maze test. **(e)** Percentage of time spent in open arms in elevated plus maze test. n=8-12 in each

group. **(f)** Percentage of open arm entries in elevated plus maze test. n=8-12 in each group. **(g)** Average heatmap in open field test. **(h)** Total distance travelled in open field test. n=8-13 in each group. **(i)** Time spent in center zone in open field test. n=8-13 in each group. **(j)** Center zone entries in open field test. n=8-13 in each group.

Supplementary Table 1. Information for patients with AD and normal controls

Case	Sex	Age (years)	Postmortem interval (min)
AD-1	M	85	930
AD-2	M	87	389
AD-3	F	84	140
AD-4	F	86	1080
AD-5	M	83	270
AD-6	M	73	570
CTR-1	M	98	220
CTR-2	M	87	300
CTR-3	F	98	277
CTR-4	M	81	675
CTR-5	M	52	1890
CTR-6	F	60	135

Note: AD = Alzheimer's disease; CTR = normal control.

Supplementary Table 2. The primers used to construct mutant plasmids

ELK1-F	cttggtaccgagctcggatccgccaccatggacccatctg
ELK1-R	gaggctgatcagcgggtttaaactcaagcgtagtcaggtacatcgtaa
P-ELK1(Ser383)-F	gcactctggatccaattgcaccccgtagtccag
P-ELK1(Ser383)-R	caattggatccagagtgctccagaaatggatgc
P-ELK1(Ser383/389)-F	attgcaccccgtgatccagccaagctctccttcc
P-ELK1(Ser383/389)-R	tggatcacggggtgcaattggatccagagtgct

Supplementary Table 3. Potential ELK1-binding sequence (WT) and corresponding mutation sequence (Mut) in the APP, BACE1, PS1 promoter

APP ELK1(1)	ccaacactaccggaagtctgga
APP ELK1(1) Mut	ccaacatgtgtactgctctgga
APP ELK1(2)	atattcaaccaggaagcagcta
APP ELK1(2) Mut	atattctgtgtactgccagcta
APP ELK1(3)	gatcttcagactgaaaatgaaa
APP ELK1(3) Mut	gatctttgtgtactgcatgaaa
APP ELK1(4)	ttccagcctcctgaaatgtgag
APP ELK1(4) Mut	ttccagtgtgtactgctgtgag
APP ELK1(5)	tggaaggaaccagaaaccatgc
APP ELK1(5) Mut	tggaagtgtgtactgcccatgc
APP ELK1(6)	acctcagggcaggaaatggcat
APP ELK1(6) Mut	acctcatgtgtactgctggcat
APP ELK1(7)	acctcagggcaggaaatggcat
APP ELK1(7) Mut	acctcatgtgtactgctggcat
APP ELK1(8)	catgcagacccggaacaaatac
APP ELK1(8) Mut	catgcatgtgtactgcaaatac
BACE1 ELK1(1)	agcctgaacccgggaggcagag
BACE1 ELK1(1) Mut	agcctgtgtgtactgcgcagag
BACE1 ELK1(2)	tgcttgaacccgggaggcggag
BACE1 ELK1(2) Mut	tgcttgtgtgtactgcgcggag
BACE1 ELK1(3)	tctccagccccggaagccggat
BACE1 ELK1(3) Mut	tctccatgtgtactgcccggat
BACE1 ELK1(4)	ccagccccgccgggagcccgcg
BACE1 ELK1(4) Mut	ccagcctgtgtactgccccgcg
PS1 ELK1(1)	cgatgtgagtcggacagtctga
PS1 ELK1(1) Mut	cgatgttgtgtactgcgtctga
PS1 ELK1(2)	ggggtggagactgaaggaacac

PS1 ELK1(2) Mut	ggggtgtgtgtactgcgaacac
PS1 ELK1(3)	tcaggccatccggatgtatacg
PS1 ELK1(3) Mut	tcaggctgtgtactgctatacg
PS1 ELK1(4)	cccaaagtgccggaattacagg
PS1 ELK1(4) Mut	cccaaatgtgtactgctacagg
PS1 ELK1(5)	acgccagagccggaaatgacga
PS1 ELK1(5) Mut	acgccatgtgtactgctgacga
PS1 ELK1(6)	ggtttcacatcggaaacaaaac
PS1 ELK1(6) Mut	ggtttctgtgtactgccaaaac
PS1 ELK1(7)	agccttggtccggaaatgctgt
PS1 ELK1(7) Mut	agcctttgtgtactgctgctgt
-10 regions of PS1 promotor	cgccagagccggaaatgacgacaacggt
-10 regions of PS1 promotor Mut	cgccagagccttaaatgacgacaacggt
